# Putative Nucleotide-Based Second Messengers in the Archaeal Model Organisms *Haloferax volcanii* and *Sulfolobus acidocaldarius*

**DOI:** 10.3389/fmicb.2021.779012

**Published:** 2021-11-22

**Authors:** Frank Braun, Alejandra Recalde, Heike Bähre, Roland Seifert, Sonja-Verena Albers

**Affiliations:** ^1^Molecular Biology of Archaea, Institute of Biology, University of Freiburg, Freiburg, Germany; ^2^Research Core Unit Metabolomics, Hannover Medical School, Hanover, Germany

**Keywords:** archaea, *Haloferax volcanii*, *Sulfolobus acidocaldarius*, cyclic nucleotides, second messengers, signaling molecules

## Abstract

Research on nucleotide-based second messengers began in 1956 with the discovery of cyclic adenosine monophosphate (3′,5′-cAMP) by Earl Wilbur Sutherland and his co-workers. Since then, a broad variety of different signaling molecules composed of nucleotides has been discovered. These molecules fulfill crucial tasks in the context of intracellular signal transduction. The vast majority of the currently available knowledge about nucleotide-based second messengers originates from model organisms belonging either to the domain of eukaryotes or to the domain of bacteria, while the archaeal domain is significantly underrepresented in the field of nucleotide-based second messenger research. For several well-stablished eukaryotic and/or bacterial nucleotide-based second messengers, it is currently not clear whether these signaling molecules are present in archaea. In order to shed some light on this issue, this study analyzed cell extracts of two major archaeal model organisms, the euryarchaeon *Haloferax volcanii* and the crenarchaeon *Sulfolobus acidocaldarius*, using a modern mass spectrometry method to detect a broad variety of currently known nucleotide-based second messengers. The nucleotides 3′,5′-cAMP, cyclic guanosine monophosphate (3′,5′-cGMP), 5′-phosphoadenylyl-3′,5′-adenosine (5′-pApA), diadenosine tetraphosphate (Ap_4_A) as well as the 2′,3′-cyclic isomers of all four RNA building blocks (2′,3′-cNMPs) were present in both species. In addition, *H. volcanii* cell extracts also contain cyclic cytosine monophosphate (3′,5′-cCMP), cyclic uridine monophosphate (3′,5′-cUMP) and cyclic diadenosine monophosphate (3′,5′-c-di-AMP). The widely distributed bacterial second messengers cyclic diguanosine monophosphate (3′,5′-c-di-GMP) and guanosine (penta-)/tetraphosphate [(p)ppGpp] could not be detected. In summary, this study gives a comprehensive overview on the presence of a large set of currently established or putative nucleotide-based second messengers in an eury- and a crenarchaeal model organism.

## Introduction

During cellular signal transduction, most external/environmental stimuli do not directly interact with their respective cellular target, but rather cause the intracellular production/release of specific small molecules, which transmit the initial signal and eventually trigger a specific cellular response. In this concept, the initial stimulus is referred to as “first messenger,” while the intracellularly transducing small molecules are called “second messengers” (Newton et al., 2016). Various second messengers have been identified and they can be grouped into four different categories according to their chemical properties: ions, gases and free radicals, lipid-based and nucleotide-based second messengers (Newton et al., 2016). Calcium (Ca^2+^) is a well-established ionic second messenger, which plays a crucial role in a plethora of eukaryotic signal transduction processes such as the excitability of neural cells, exocytosis, motility, apoptosis, and transcription (Clapham, 2007). A well-characterized example of a gaseous second messenger is nitric oxide (NO). Molecules of this gas are involved in various prokaryotic and eukaryotic signal transduction pathways, such as the regulation of the mammalian nervous system and bacterial quorum sensing and biofilm formation (Zhou and Zhu, 2009; Nisbett and Boon, 2016). Examples of lipid-based second messengers are diacylglycerol or ceramide, which are involved in various eukaryotic signal cascades (Liscovitch and Cantley, 1994; Hilgemann et al., 2018). The most diverse category of second messengers consists of nucleotide-based signaling molecules. These second messengers can either be based on a single (mono-nucleotide-based), two (di-nucleotide-based), or several (oligo-nucleotide-based) nucleotide molecules. Table 1 shows an overview on the majority of currently established or putative nucleotide-based second messengers, including their presence and some examples of their functions in eukaryotes and bacteria.

**TABLE 1:** Overview on occurrence and exemplary function(s) in eukaryotes and bacteria of all signaling nucleotides addressed in this study.

Nucleotide(s)	Presence in eukaryotes	Exemplary functions in eukaryotes	Presence in bacteria	Exemplary functions in bacteria
**Mono-nucleotide-based signaling molecules**				
3′,5′-cAMP	In a multitude of uni- and multicellular species (Shemarova, 2009; Gancedo, 2013; Blanco et al., 2020)	• Regulation of carbohydrate metabolism (Sutherland and Wosilait, 1956; Berthet et al., 1957; Rall and Sutherland, 1958) • Synaptic transmission (Marx, 1972; Maiellaro et al., 2016) • Seed germination (Uematsu and Fukui, 2008) • Chemotaxis (Escalante et al., 1997)	In species of various phyla (Botsford and Harman, 1992)	• Catabolite repression (Crasnier, 1996) • Infection and host colonialization (McDonough and Rodriguez, 2011) • Biofilm formation (Liu et al., 2020)

3′,5′-cGMP	Mainly found in ciliated eukaryotes (Johnson and Leroux, 2010)	• Phototransduction (Stryer, 1986) • Smooth muscle relaxation (Rybalkin et al., 2003) • Osmoregulation (Kuwayama et al., 1996)	Identified in f.i.: • Cyanobacteria (Cadoret et al., 2005) •α-proteobacteria (Marden et al., 2011) •γ-proteobacteria (An et al., 2013)	• UV-stress adaption (Cadoret et al., 2005) • Cyst formation (Marden et al., 2011) • Biofilm and virulence (An et al., 2013)

3′,5′-cCMP 3′,5′-cUMP	Using modern mass spectrometry detected in various mammalian cell lines and different organs (Burhenne et al., 2011; Bähre et al., 2015). Additionally also found in zebrafish (Dittmar et al., 2015) and cUMP in a plant (Hartwig et al., 2014)	• In general functions are currently mostly unknown (Seifert, 2017) • Both can activate some cAMP/cGMP effectors *in vitro* (Wolter et al., 2011; Zong et al., 2012) • cCMP potentially involved in processes such as tissue development and cell proliferation, immune responses modulation and platelet aggregation (Bloch et al., 1974; Anderson, 1982; Desch et al., 2010)	• Specific cytidylate and uridylate cyclases just recently discovered in species of various phyla (Tal et al., 2021) • Synthesis stimulated by phage infection; activate downstream defense mechanisms (Tal et al., 2021) • Bacterial toxins [e.g., ExoY from *P. aeruginosa* (Beckert et al., 2014), CyaA from *Bordetella pertussis* (Göttle et al., 2010), edema factor from *Bacillus anthracis* (Göttle et al., 2010)] have been demonstrated to be capable of forming cCMP and cUMP	

3′,5′-cIMP	Identified in isolated porcine coronary arteries (Chen et al., 2014)	• In general functions are currently mostly unknown (Leung et al., 2015) • Involved in hypoxia−induced constriction of porcine coronary arteries (Chen et al., 2014; Nan et al., 2020)	• Only very few data available • Detected in *Corynebacterium murisepticum* (Newton et al., 1998); specificity of the used method is however questioned (Seifert, 2015)	

3′,5′-cTMP 3′,5′-cXMP	Not yet detected in any biological samples using modern and sensitive mass spectrometry techniques (Beste and Seifert, 2013) cXMP can be formed by purified guanylate cyclase and activate certain cAMP effectors *in vitro* (Wolter et al., 2011; Beste et al., 2012)			

2′,3′-cNMPs	Identified in several mammalian cell lines (Ren et al., 2009; Pabst et al., 2010; Bähre and Kaever, 2014), different organs (Jia et al., 2014) and plant tissue (Van Damme et al., 2014)	• Originate from mRNA degradation by transphosphorylation (Thompson et al., 1994) or RNA cyclase activity (Shigematsu et al., 2018) • Actual utilization as second messengers currently unknown; reporting of tissue damage as possible function (Jackson, 2011, 2017; Van Damme et al., 2014)	Identified in f.i.: • *Pseudomonas fluorescens* (Bordeleau et al., 2014) • *E. coli* (Fontaine et al., 2018)	• In *E. coli* originating from RNase I-dependent RNA degradation (Fontaine et al., 2018) or RNA cyclase activity (Genschik et al., 1997) • Levels of 2′,3′-cNMPs influenced the biofilm formation of *E. coli* (Fontaine et al., 2018)

(p)ppGpp	• For a long time believed to be absent in eukaryotes, with the notable exception of chloroplasts (Tozawa and Nomura, 2011) • Recently found in *Drosophila melanogaster* and human cell lines (Ito et al., 2020) • Altered levels caused metabolic changes and cell death in *D. melanogaster* (Ito et al., 2020)		In species of various phyla (Atkinson et al., 2011)	• Stress signaling related alarmones (Cashel and Gallant, 1969; Lazzarini et al., 1971) • Synthesis triggered by diverse metabolic or physical stresses (Cashel and Gallant, 1969; Gallant et al., 1977; Flärdh et al., 1994; Spira et al., 1995; Vinella et al., 2005; Battesti and Bouveret, 2006; Hood et al., 2016; Tarusawa et al., 2016) • Globally regulates gene transcription in the context of the stringent response (Magnusson et al., 2005)

**Di-nucleotide-based signaling molecules**				
3′,5′-c-di-GMP	• Synthesizing and degrading enzymes bioinformatically predicted in lower eukaryotes (Römling et al., 2013) • Synthesis confirmed in social amoebae of the class of Dictyostelia with a function for stalk cell differentiation (Chen and Schaap, 2012) • Recognized by human innate immune system (Burdette et al., 2011)		In species of various phyla (Römling et al., 2013)	• Involvement in various physiological functions observed (Römling et al., 2005) • Major functions: Transition from motile to sessile lifestyle (Wolfe and Visick, 2008; Valentini and Filloux, 2016), virulence (Valentini and Filloux, 2019)

3′,5′-c-di-AMP	• Currently believed to be absent from eukaryotic cells (He et al., 2020) • Recognized by human innate immune system (Barker et al., 2013)		In species of various phyla (Corrigan and Gründling, 2013)	• Involvement in several physiological functions observed (Fahmi et al., 2017) • Major function: Regulation of cellular osmotic homeostasis (Stülke and Krüger, 2020)

2′,3′-cGAMP	Currently believed to only be present in metazoa (Kranzusch, 2019)	• Endogenous activator of the innate immune system leading to type-I interferon production (Wu et al., 2013; Zhang et al., 2013)	Isomer containing the atypical 2-′5′ phosphodiester linkage currently believed to be exclusively found in metazoa (Davies et al., 2012; Ablasser et al., 2013; Diner et al., 2013; Gao et al., 2013; Li et al., 2019)	

3′,3′-cGAMP	Produced by some lower metazoa like the anemone *Nematostella vectensis* (Kranzusch et al., 2015)	• Same function as 2′,3′-cGAMP as endogenous activator of the innate immune system (Kranzusch et al., 2015)	Synthesizing enzymes identified in various phyla (Whiteley et al., 2019)	• Biofilm formation and motility (Li et al., 2019) • Protection against viral infection (Severin et al., 2018; Cohen et al., 2019)

3′,2′-cGAMP	Very recently identified in *D. melanogaster*; synthesizing enzymes also conserved in metazoa (Holleufer et al., 2021; Slavik et al., 2021)	• Very similar function as 2′,3′-cGAMP as an endogenous activator of innate immune response (Holleufer et al., 2021; Slavik et al., 2021)	Currently no data available on the existence in bacteria; Presence of the atypical 2-′5′ phosphodiester linkage suggest its absence in bacteria (see 2′,3′-cGAMP above)	

5′-pGpG 5′-pApA	• Actual function as second messenger currently unclear • In bacteria, both are the degradation product of 3′,5′-c-di-GMP and 3′,5′-c-di-AMP, respectively (Rao et al., 2010; Stelitano et al., 2013) • Both have the capability to bind to some targets which are regularly binding the respective, unhydrolyzed c-di-NMP (Smith et al., 2012; Stelitano et al., 2013; Bowman et al., 2016; Kuipers et al., 2016) • As a nanoRNA, both have the potential to affect gene transcription on the level of transcription initiation (Goldman et al., 2011; Nickels and Dove, 2011)			

Ap_4_A	In a multitude of uni- and multicellular species (Plesner and Ottesen, 1980; Flodgaard and Klenow, 1982; Garrison and Barnes, 1984; McLennan and Prescott, 1984)	• Potential stress signaling related alarmone (Brevet et al., 1985; Baltzinger et al., 1986; Coste et al., 1987; Garrison et al., 1989) • In metazoa: regulatory effects on the cardiovascular and immune system and neuronal signal transduction (Vahlensieck et al., 1999; Miras-Portugal et al., 2003; Lee et al., 2004)	Synthesizing and hydrolyzing enzymes present in various phyla (Ferguson et al., 2020)	• Potential stress signaling related alarmone (Lee et al., 1983; Pálfi et al., 1991; Kimura et al., 2017) • Cellular development (Nishimura et al., 1997; Kimura et al., 2017) • Biofilm formation (Monds et al., 2010)

**Oligo-nucleotide-based signaling molecules**				

cOA	According to current knowledge absent in eukaryotes		In species utilizing a type III CRISPR system (Kazlauskiene et al., 2017; Niewoehner et al., 2017)	• Produced upon presence of invader RNA (Kazlauskiene et al., 2017; Niewoehner et al., 2017) • Activate effectors leading to invader RNA/DNA degradation (Kazlauskiene et al., 2017; Niewoehner et al., 2017; Koonin and Makarova, 2018)
				

*3′,5′-cAMP, 3′,5′-cyclic adenosine monophosphate; 3′,5′-cGMP, 3′,5′-cyclic guanosine monophosphate; 3′,5′-cCMP, 3′,5′-cyclic cytidine monophosphate; 3′,5′-cUMP, 3′,5′-cyclic uridine monophosphate; 3′,5′-cIMP, 3′,5′-cyclic inosine monophosphate; 3′,5′-cTMP, 3′,5′-cyclic thymidine monophosphate; 3′,5′-cXMP, 3′,5′-cyclic xanthosine monophosphate; 2′,3′-cNMPs, 2′,3′-cyclic isomers of nucleotides with N here: adenosine, guanosine, cytidine or uridine; (p)ppGpp, guanosine (penta-)/tetraphosphate; 3′,5′-c-di-GMP, 3′,5′-cyclic diguanosine monophosphate; 3′,5′-c-di-AMP, 3′,5′-cyclic diadenosine monophosphate; 2′,3′-cGAMP, 2′,3′-cyclic guanosine monophosphate-adenosine monophosphate (cyclic [G(2′,5′)pA(3′,5′)p]); 3′,3′-cGAMP, 3′,3′-cyclic guanosine monophosphate-adenosine monophosphate (cyclic [G(3′,5′)pA(3′,5′)p]); 3′,2′-cGAMP, 3′,′-cyclic guanosine monophosphate-adenosine monophosphate (cyclic [G(3′,5′)pA(2′,5′)p]); 5′-pGpG, 5′-phosphoguanylyl-3′,5′-guanosine; 5′-pApA, 5′-phosphoadenylyl-3′,5′-adenosine; Ap_4_A, diadenosine tetraphosphate; cOA, cyclic oligoadenylate (with n = 3–6); CRISPR, Clustered Regularly Interspaced Short Palindromic Repeats; f.i., for instance.*

The information summarized in Table 1 originates from bacteria and eukaryotes. For archaea, only very limited information about the occurrence and the physiological functions of nucleotide-based second messengers is currently available. Until now, only the presence of 3′,5′-cAMP, 3′,5′-c-di-AMP and cyclic oligo adenylate (cOA) has been reported in archaea. 3′,5′-cAMP was identified in the euryarchaea *Haloferax volcanii* and *Methanothermobacter thermoautotrophicus* and in the crenarchaea *Saccharolobus solfataricus* (previously known as *Sulfolobus solfataricus*) (Leichtling et al., 1986). Additionally, in the euryarchaeon *Halobacterium salinarum* the levels of 3′,5′-cAMP were shown to fluctuate during the cell cycle (Baumann et al., 2007). Analogous to its reported function in bacteria, cOA was shown to be involved in type III CRISPR system mediated immunity in the crenarchaeon *Sa. solfataricus* (Rouillon et al., 2018). In *H. volcanii*, 3′,5′-c-di-AMP was shown to be essential and has been implicated in osmoregulation (Braun et al., 2019). Noteworthy, analysis of the presence of the alarmone (p)ppGpp using radioisotope-labeling approaches in a few archaeal species suggested the absence of this signaling nucleotide (Beauclerk et al., 1985; Cimmino et al., 1993; Scoarughi et al., 1995; Cellini et al., 2004). For all other nucleotide-based second messengers there is, to the best of our knowledge, currently no data available whether or not they are produced in archaea. To study the presence of nucleotide-based second messengers in archaea, cell extracts of the euryarchaeal model organism *H. volcanii* and the crenarchaeal model organism *S. acidocaldarius* were analyzed using liquid chromatography tandem mass spectrometry (LC−MS/MS) for the presence of representatives of mono-, di-, and oligo-nucleotide-based second messengers. These measurements unveiled that *H. volcanii* cells contain, besides the already known 3′,5′-cAMP and 3′,5′-c-di-AMP, detectable levels of 3′,5′-cGMP, 3′,5′-cCMP, 3′,5′-cUMP, 2′,3′-cAMP, 2′,3′-cGMP, 2′,3′-cCMP, 2′,3′-cUMP, 5′-pApA and Ap_4_A. Compared to that, *S. acidocaldarius* cells contained a reduced variety of nucleotides. Besides all four 2′,3′-cNMPs, only 3′,5′-cAMP, 3′,5′-cGMP, Ap_4_A and very minor amounts of 5′-pApA could be detected. The well-established bacterial second messenger 3′,5′-c-di-GMP, the alarmone (p)ppGpp as well as all three physiologically appearing isomers of cGAMP (2′,3′-cGAMP, 3′,3′-cGAMP, and 3′,2′-cGAMP) could not be detected, suggesting their absence in *H. volcanii* and *S. acidocaldarius* when grown under standard laboratory conditions. The same applies for 3′,5′-cTMP, 3′,5′-cIMP, 3′,5′-cXMP, 5′-pGpG and cOA (*n* = 4; c-tetra-AMP), which were all not present in the examined *H. volcanii* and *S. acidocaldarius* cell extracts.

Taken together, the results of this study show that the nucleotide-based second messenger pools of *H. volcanii* and *S. acidocaldarius* contain several signaling molecules, whose presence in archaea has not been shown so far. Assuming that many other euryarchaeal and crenarchaeal species make use of similar nucleotide-based second messenger pools, these results offer important leads to further investigate the role and importance of these nucleotides for these organisms.

## Materials and Methods

Unless stated otherwise all chemicals were purchased from Carl Roth.

### Strains and Growth Conditions

*H. volcanii* strain H26 was grown in selective CA medium (Allers et al., 2004) (0.5 g/L Bacto*™* Casamino acids; pH 7.2 adjusted with KOH) modified with an expanded trace element solution (referred to as CAB) (Duggin et al., 2015). Cells were grown at 45°C in liquid medium while rotating (volumes up to 5 ml) or shaking (volumes > 5 ml).

*S. acidocaldarius* strain MW001 was grown in basal Brock medium (pH 3.5) (Brock et al., 1972) supplemented with 0.1% (w/v) NZ-amine (Sigma) and 0.2% (w/v) dextrin. Cells were grown at 75°C in liquid medium while shaking.

Since both strains are auxotroph for uracil (H26, Δ*pyrE2* and MW001, Δ*pyrEF*), growth media were supplemented with uracil (Sigma) at a defined concentration (50 μg/ml for H26; 10 μg/ml for MW001). Further details on H26 and MW001 are listed in Supplementary Table 1.

### Nucleotide Extraction From *Haloferax volcanii* and *Sulfolobus acidocaldarius* Cells

The extraction of nucleotides from total cell pellets of *H. volcanii* and *S. acidocaldarius* cells was performed as described previously (Spangler et al., 2010; Braun et al., 2019). Briefly, H26 was grown in 340 ml CAB + uracil, MW001 in 250 ml supplemented Brock + uracil. Samples were taken during exponential growth and at the beginning of the stationary phase. For nucleotide extraction from exponentially growing cultures 25 ml were harvested, for stationary grown cultures 15 ml were harvested. For each nucleotide sample, an additional 2 ml aliquot of each culture was harvested for the determination of the total protein content [bicinchoninic acid (BCA) Protein Assay Macro Kit (Serva)]. Cell pellets were snap−frozen in liquid nitrogen. Unless mentioned otherwise the experiments were performed as three biological replicates with three technical replicates each. The cell pellets were resuspended in 300 μl extraction solution [acetonitrile/methanol/water (ultrapure): 2:2:1 (v/v/v)]. Resuspended pellets were incubated on ice for 15 min followed by a heating step at 95°C for 10 min. After cooling on ice, the solution was centrifuged at 21,100 × *g* for 10 min at 4°C. The resulting supernatant was transferred to a fresh vial. The extraction was repeated two times (three extraction steps in total) with 200 μl fresh extraction solution, omitting the heating step. The supernatants were combined and stored overnight at -20°C to precipitate proteins. To remove precipitates, the samples were centrifuged again (10 min, 4°C; 21,100 × *g*) and the supernatant was transferred to a fresh vial. Final extracts were desiccated using a vacuum concentrator (Eppendorf) at 45°C.

### Nucleotide Extraction From *Haloferax volcanii* Cell Lysate via Solid Phase Extraction

Nucleotide extracts from total cell pellets of *H. volcanii* could not be analyzed for their (p)ppGpp content due to certain specific fragmentation patterns of unknown molecular species overlapping with the internal (p)ppGpp standard. Therefore, extraction of (p)ppGpp was performed using a solid phase extraction approach as described previously (Ihara et al., 2015). For solid phase extraction from exponentially growing cultures 15 ml were harvested, for stationary grown cultures 9 ml were harvested. For each sample, an additional 2 ml aliquot of each culture was harvested for the determination of the total protein content (BCA assay). Growth of H26 and time points of sample acquisition were as described above. Cell pellets were snap−frozen in liquid nitrogen and subsequently resuspended in 2 ml ultrapure water on ice. Resuspended pellets were lysed by the addition of formic acid (to a final concentration of 1 M) and incubated for 1 h on ice. The cell lysate was mixed with 2 ml ammonium acetate (pH 4.5) and centrifuged for 5 min at 4°C and 3,000 × *g* to remove cell debris. The lysate was further purified on an OASIS Wax cartridge 1 cc (Waters) using centrifugation steps of 1 min at 4,300 × *g* at 4°C. The cartridge was equilibrated with 1 ml methanol followed by 1 ml ammonium acetate (pH 4.5), the lysate was loaded in four consecutive loading steps of 1 ml, the cartridge was washed with 1 ml ammonium acetate (pH 4.5) followed by 1 ml methanol and the sample was eluted with 1 ml elution solvent [water (ultrapure)/methanol/ammonium hydroxide (25% (w/v)): 7:2:1 (v/v/v)]. The elution fractions were desiccated using a vacuum concentrator (Eppendorf) at 45°C.

### Quantification of Nucleotides From Cell Extracts by Liquid Chromatography With Tandem Mass Spectrometry

Desiccated nucleotide extracts were resuspended in 200 μl water, centrifuged and diluted 1:2 with the respective standard solution (containing stable isotope labeled nucleotides as well as 100 mg/ml Tenofovir as internal standards) and analyzed by a LC−MS/MS method.

Cyclic di-nucleotides were analyzed as described previously (Rao et al., 2010; Bähre and Kaever, 2017). Chromatographic separation was performed by reversed phase chromatography on a C18-column (Nucleodur Pyramid C18 3 μ 50 × 3 mm; Macherey-Nagel; Germany), using water containing 10 mM ammonium acetate and 0.1% acetic acid as eluent A and pure methanol as eluent B, using the following gradient: 0–4 min 0% B, 4–7.3 min 0–10% B, 7.3–8.3 min 10% B, 8.3–11 min 10–30% B, 11–13 min 0% B. The flow rate was 600 μl/min. Mass spectrometric analysis was performed on a tandem mass spectrometer (API4000; MA, United States) performing selected reaction monitoring (SRM). The mass spectrometer was equipped with an electrospray ionization source (ESI) and ionization was performed in positive mode for all analytes.

Cyclic nucleotides, were analyzed as described previously (Bähre and Kaever, 2014). Chromatographic separation was performed by reversed phase chromatography on a C18-column, using methanol-water [3:97 (v/v)] containing 50 mM ammonium acetate and 0.1% (v/v) acetic acid as eluent A and methanol-water [97:3 (v/v)] containing 50 mM ammonium acetate and 0.1% (v/v) acetic acid as eluent B, using the following gradient: 0–5 min 0–50% B and 5–8 min 0% B. The flow rate was 500 μl/min. Cyclic nucleotides were analyzed on a QTRAP 5500 (Sciex, MA, United States). Ionization was achieved with an ESI in positive mode. In SRM mode 3′,5′-cNMPs and the 2′,3′-cNMPs show the same mass transitions due to their high structural similarity. However, these analytes were clearly identified by their different retention times.

Ap_4_A, ppGpp, and pppGpp were analyzed as described previously (Schäfer et al., 2020). Chromatographic separation was performed on a Hypercarb column (30 × 4.6 mm, 5 μm particle size; Thermo Fisher, Scientific MA, United States) using 10 mM ammonium acetate (pH 10) as eluent A and acetonitril as eluent B, using an 8 min gradient from 4 to 60% B. The flow rate was 600 μl/min. All analytes were detected by LC-MS/MS on a QTRAP 5500 (Sciex MA, United States). Ionization of analytes was achieved with an ESI in positive ion mode and SRM was used for analyte detection.

For all used LC-MS/MS methods, the control of the LC and the mass spectrometers as well as data sampling was performed using Analyst software (version 1.7 Sciex, MA, United States). For quantification, calibration curves were created by plotting peak area ratios of the analyte, and the internal standard vs. the nominal concentration of the 10 calibrators. The calibration curve was calculated using quadratic regression and 1/× weighing.

The measured concentration of each nucleotide was normalized for each cell extract sample to the total protein concentration of the respective sample.

## Results and Discussion

### *Haloferax volcanii* and *Sulfolobus acidocaldarius* Cells Grown Under Standard Conditions Contain Several Mono-Nucleotide-Based (Putative) Second Messengers

We set out to identify nucleotide-based (putative) signaling molecules in the euryarchaeal and crenarchaeal model organisms *H. volcanii* and *S. acidocaldarius*. In a first step, we screened for mono-nucleotide-based (putative) second messengers. The presence of 3′,5′-cAMP in *H. volcanii* cells has been described 35 years ago (Leichtling et al., 1986). Except for 3′,5′-cAMP, the presence of other mono-nucleotide-based (putative) second messengers, like other 3′,5′-cyclic nucleotides (3′,5′-cNMPs), 2′,3′-cNMPs or (p)ppGpp, has not been detected in any archaeal species so far. No reports are available on the presence of 3′,5′-cAMP in *S. acidocaldarius*, but the closely related species *Sa. solfataricus* has been shown to produce 3′,5′-cAMP (Leichtling et al., 1986), implying that this nucleotide is most likely also present in *S. acidocaldarius*.

**FIGURE 1:**
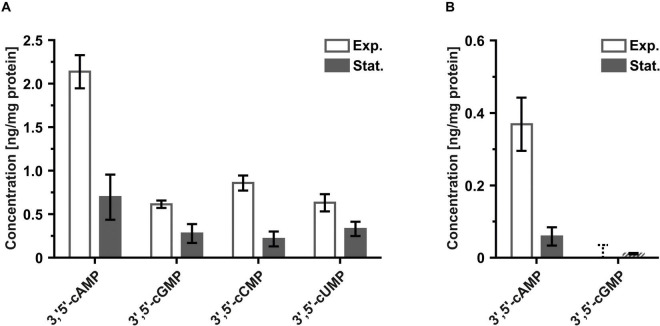
Levels of 3′,5′-cNMPs detected for exponentially and stationary growing (A) *H. volcanii* H26 and (B) *S. acidocaldarius* MW001 cells normalized to the protein content of each sample. Values of regular columns represent the mean of three biological replicates. Error bars of regular columns indicate standard deviation of three biological replicates. Dashed columns indicate the presence of one or more technical replicates with measured nucleotide levels below the lowest limit of quantification (LLOQ). Values of dashed columns represent the mean of all technical replicates with nucleotide levels ≥ LLOQ. Error bars of dashed columns indicate standard deviation of all technical replicates with nucleotide levels ≥ LLOQ. Dashed error bars indicate absence of a single technical replicate with measured nucleotide levels ≥ LLOQ. Values of dashed error bars represent the highest calculated value of all analyzed technical replicates with measured nucleotide levels < LLOQ. The exact nucleotide concentrations are summarized in Table 2 (Exp., exponential; Stat., stationary).

*H. volcanii* strain H26 and *S. acidocaldarius* strain MW001 were grown under standard laboratory conditions. These cultures were used to obtain cell material from the exponential and stationary growth phases for nucleotide extraction (Supplementary Figure 1). Extraction of nucleotides from the cell extracts followed by LC-MS/MS not only confirmed the presence of 3′,5′-cAMP in these two species (Figures 1A,B) but also revealed that 3′,5′-cGMP, 3′,5′-cCMP and 3′,5′-cUMP are present in *H. volcanii* as well (Figure 1A). These four 3′,5′-cNMPs showed increased levels in exponentially growing *H. volcanii* cells compared to stationary cells (Figure 1A). Similar observations were made for 3′,5′-cAMP in exponentially growing *S. acidocaldarius* cells, which contained higher amounts of this cyclic nucleotide compared to stationary growth (Figure 1B). 3′,5′-cCMP and 3′,5′-cUMP were not detected in any *S. acidocaldarius* sample (Figure 1B), suggesting that these nucleotides, at least under the tested growth conditions, are not synthesized by this crenarchaeon. 3′,5′-cGMP was the only other 3′,5′-cNMP detected in *S. acidocaldarius* (Figure 1B). However, the levels of 3′,5′-cGMP were low in both tested conditions. Five of nine replicates from the stationary culture (originating from three biological replicates with three technical replicates each) contained sufficient amounts of 3′,5′-cGMP for a quantitative analysis. In all other samples from *S. acidocaldarius* (four replicates from stationary cells and all nine replicates from exponentially growing cells), 3′,5′-cGMP could be detected as well, but at levels which did not allow for a valid quantification. Therefore no reliable 3′,5′-cGMP level could be calculated for this crenarchaeon.

With (some) 3′,5′-cNMPs being present in *H. volcanii* and *S. acidocaldarius* the question arises by which enzymes they are produced. Synthesis of 3′,5′-cAMP *in vivo* is achieved by adenylate cyclases (ACs) of which six different classes (I–VI) are currently known (Khannpnavar et al., 2020). Very few predicted archaeal ACs fall into class III (Bassler et al., 2018) [cluster of orthologous groups (COG) 2114], while the vast majority belongs to class IV (COG1437), which is characterized by a CYTH (CyaB, thiamine triphosphatase) domain. *H. volcanii* as well as *S. acidocaldarius* each contain a single gene encoding for a putative class IV AC (*HVO*_1648 and *Saci*_0718). The *S. acidocaldarius* gene product of *Saci*_0718 has recently, however, been demonstrated not to function as a cyclase but as a phosphohydrolase of the triphosphate tunnel metalloenzyme (TTM) family (Vogt et al., 2021). An in this context performed systematic sequence similarity network analysis of the CYTH superfamily unveiled that actual class IV ACs only account for a small subgroup, which is entirely of bacterial origin (Vogt et al., 2021). This is in accordance with the observation that a *H. volcanii* deletion mutant lacking *HVO*_1648 has unchanged 3′,5′-cAMP levels (preliminary data). Together, these observations imply that most archaea are likely to synthesize 3′,5′-cAMP with a yet unknown new class of ACs, which is structurally different from the currently established ones.

Enzymes synthesizing 3′,5′-cGMP, called guanylate cyclases, have been characterized from bacteria (Marden et al., 2011; An et al., 2013) and eukaryotes (Kang et al., 2019). Protein BLAST searches using the sequences of the guanylate cyclase domains of such enzymes (Marden et al., 2011; An et al., 2013; Kang et al., 2019) against the translated protein databases of species of the genera *Haloferax* and *Sulfolobus* yielded no results with significant similarity. This finding is supported by the fact that the COG2114 (adenylate and guanylate cyclase catalytic domain) neither contains a homolog for *H. volcanii* nor for *S. acidocaldarius*. All these observations might imply that 3′,5′-cGMP in *H. volcanii* and S. *acidocaldarius* is not generated by a classic guanylate cyclase.

3′,5′-cCMP and 3′,5′-cUMP were just recently identified to play an important role as signaling molecules in prokaryotic phage-defense systems (Tal et al., 2021). A recently performed phylogenetic analysis of proteins containing pyrimidine cyclase domains revealed that these types of enzymes are also found in few euryarchaeal species (Tal et al., 2021). However, BLAST searches of these identified putative euryarchaeal pyrimidine cyclases in the proteome of *H. volcanii* yielded no hit. The same applies when experimentally characterized bacterial pyrimidine cyclases (Tal et al., 2021) were used as template. This observation might suggest the existence of additional and more distantly to the recently discovered pyrimidine cyclases related types of specific cytidylate/uridylate cyclases. Nevertheless, since guanylate and adenylate cyclases were identified or are assumed to be capable of not only producing their respective intrinsic products but also 3′,5′-cCMP and 3′,5′-cUMP under certain conditions (Beste et al., 2012; Bähre et al., 2014; Seifert, 2017), it is possible that the detected 3′,5′-cCMP and 3′,5′-cUMP originate from a divergent enzymatic activity of these two types of cyclases.

In addition to 3′,5′-cAMP, 3′,5′-cGMP, 3′,5′-cCMP, and 3′,5′-cUMP, the cell extracts from both species were also checked for the presence of 3′,5′-cTMP, 3′,5′-cIMP, and 3′,5′-cXMP, however, none of these cyclic nucleotides could be detected (Table 2). Only very few studies show the presence 3′,5′-cIMP in biological systems (Newton et al., 1998; Chen et al., 2014), with some of these reports called into question when it comes to the specificity of the detection method used (Seifert, 2015). Therefore, it is difficult to speculate whether the absence of 3′,5′-cIMP observed in *H. volcanii* and *S. acidocaldarius* indicates a general absence of this nucleotide or an absence under the standard conditions used in this study. The absence of 3′,5′-cTMP and 3′,5′-cXMP in cell extracts from both model organisms is in line with the fact that these cyclic nucleotides have not been unequivocally identified in any living cell yet.

**TABLE 2:** Summary of measured and detected mono-nucleotide-based (putative) second messengers.

Organism	*H. volcanii*		*S. acidocaldarius*	
Molecule	Levels during exponential growth [ng/mg protein]	Levels during stationary growth [ng/mg protein]	Levels during exponential growth [ng/mg protein]	Levels during stationary growth [ng/mg protein]
*3*′,*5*′*-cAMP*	2.14 ± 0.19	0.70 ± 0.26	0.37 ± 0.07	0.06 ± 0.03
*3*′,*5*′*-cGMP*	0.61 ± 0.04	0.28 ± 0.11	≥ 0	≤ 0.01 ± 0.002
*3*′,*5*′*-cCMP*	0.86 ± 0.09	0.22 ± 0.08	n.d.	n.d.
*3*′,*5*′*-cUMP*	0.63 ± 0.10	0.33 ± 0.08	n.d.	n.d.
*3*′,*5*′*-cTMP*	n.d.	n.d.	n.d.	n.d.
*3*′,*5*′*-cIMP*	n.d.	n.d.	n.d.	n.d.
*3*′,*5*′*-cXMP*	n.d.	n.d.	n.d.	n.d.
*2*′,*3*′*-cAMP*	50.03 ± 3.70	32.23 ± 5.86	1.65 ± 0.16	1.13 ± 0.31
*2*′,*3*′*-cGMP*	36.33 ± 2.66	21.91 ± 4.80	0.92 ± 0.10	0.85 ± 0.25
*2*′,*3*′*-cCMP*	25.21 ± 1.99	18.72 ± 3.02	0.36 ± 0.18	0.53 ± 0.15
*2*′,*3*′*-cUMP*	0.84 ± 0.19	0.66 ± 0.17	n.d.	≤ 0.04 ± 0.006
*ppGpp*	n.d.	n.d.	n.d.	n.d.
*pppGpp*	n.d.	n.d.	n.d.	n.d.

*(**±**, gives standard deviation; n.d., not detectable; ≤, average of all technical replicates ≥ LLOQ; ≥ 0, nucleotide detected but all technical replicates < LLOQ).*

**FIGURE 2:**
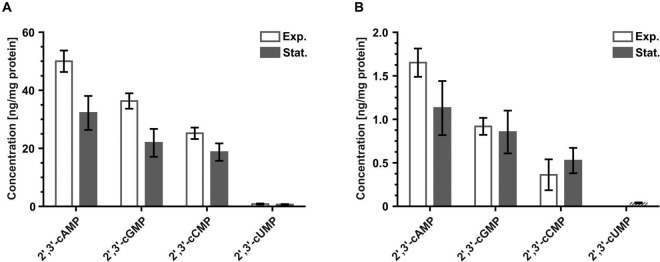
Levels of 2′,3′-cNMPs detected for exponentially and stationary growing **(A)** *H. volcanii* H26 and **(B)** *S. acidocaldarius* MW001 cells normalized to the protein content of each sample. Values of regular columns represent the mean of three biological replicates. Error bars of regular columns indicate standard deviation of three biological replicates. Dashed columns indicate presence of one or more technical replicates with measured nucleotide levels below the lowest limit of quantification (LLOQ). Values of dashed columns represent the mean of all technical replicates with nucleotide levels ≥ LLOQ. Error bars of dashed columns indicate standard deviation of all technical replicates with nucleotide levels ≥ LLOQ. The exact nucleotide concentrations are summarized in Table 2 (Exp., exponential; Stat., stationary).

Additionally to 3′,5′-cNMPs, *H. volcanii* and *S. acidocaldarius* cell extracts were also analyzed for the presence of 2′,3′-cNMPs. All four examined 2′,3′-cNMPs, namely 2′,3′-cAMP, 2′,3′-cGMP, 2′,3′-cCMP and 2′,3′-cUMP, were present in both species in samples from at least one of the two tested growth stages (Figures 2A,B). For *H. volcanii* cell extracts, the measured concentrations of 2′,3′-cAMP, 2′,3′-cGMP, and 2′,3′-cCMP (Figure 2A) were much higher than the ones of the corresponding 3′,5′-isomer (Figure 1A). Only 2′,3′-cUMP was present at concentrations similar to 3′,5′-cUMP. Similar to the 3′,5′-cNMPs, the concentrations of 2′,3′-cNMPs in *H. volcanii* generally increased during exponential growth (Figure 2A). In *S. acidocaldarius* extracts, 2′,3′-cNMP levels were in the same range for exponential and stationary growth (Figure 2B). Similarly, to what was observed for the 2′,3′-cUMP levels of *H. volcanii*, levels of this 2′,3′-cyclic nucleotide in *S. acidocaldarius* were also considerably lower in comparison to the other three 2′,3′-cNMPs. Of all stationary samples, only three technical replicates (out of nine in total) contained quantifiable amounts of 2′,3′-cUMP, whereas samples of exponentially growing *S. acidocaldarius* cells did not contain any 2′,3′-cUMP. Production of the detected 2′,3′-cNMPs in both species has most likely to be linked to the process of RNA-degradation, a major source of 2′,3′-cNMPs in eukaryotes and bacteria (Thompson et al., 1994; Fontaine et al., 2018), and/or to the activity of homologs of certain RNA cyclases/ligases, which are also known to form 2′,3′-cyclic phosphates at the 3′-ends of RNAs (Shigematsu et al., 2018). As currently no distinct function as second messenger is ascribed to any 2′,3′-cNMP it appears likely that they are not used as such in *H. volcanii* and *S. acidocaldarius* as well. Still, it also cannot be excluded that they may act in some yet to be discovered signaling network.

Next to 3′,5′-cNMPs and 2′,3′-cNMPs, the cell extracts were also analyzed for the presence of the alarmone ppGpp and its precursor pppGpp. Analysis of extracts from *S. acidocaldarius* did not detect any (p)ppGpp at the tested conditions (Table 2). Since cell extracts of *H. volcanii* contained substances that interfered with the (p)ppGpp internal standard signal, an alternative solid phase extraction protocol was used (see section “Materials and Methods”). For these extracts the internal (p)ppGpp standard signal was unaffected and the corresponding measurements revealed that neither ppGpp nor pppGpp was present in the *H. volcanii* samples (Table 2). These observations are in accordance with former studies examining the occurrence of (p)ppGpp in both species which showed that this alarmone was not produced, even when cells were subjected to stress factors such as starvation (Scoarughi et al., 1995; Cellini et al., 2004). In line with this, a study on the distribution of (p)ppGpp synthetases and hydrolases across the tree of life suggests that *H. volcanii* and *S. acidocaldarius* do not contain any of these enzymes (COG0317 contains no hit for both organisms) and that they are in general only very rarely found in archaea (Atkinson et al., 2011).

### Cyclic Diadenosine Monophosphate, 5′-Phosphoadenylyl-3′,5′-Adenosine, and Diadenosine Tetraphosphate Are the Only Di-Nucleotide-Based (Putative) Second Messengers Measured in at Least One of the Two Archaeal Model Organisms

**FIGURE 3:**
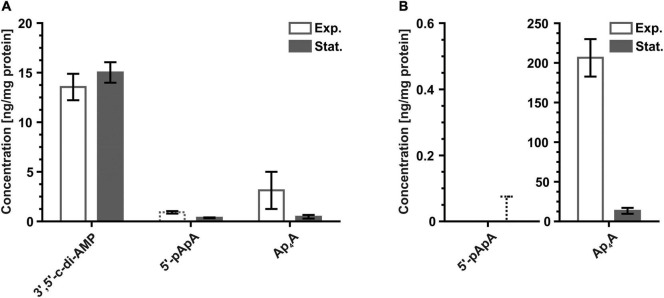
Levels of di-nucleotide-based (putative) second messengers detected for exponentially and stationary growing **(A)** *H. volcanii* H26 and **(B)** *S. acidocaldarius* MW001 cells normalized to the protein content of each sample. Values of regular columns represent the mean of three biological replicates. Error bars of regular columns indicate standard deviation of three biological replicates. Dashed columns indicate presence of one or more technical replicates with measured nucleotide levels below the lowest limit of quantification (LLOQ). Values of dashed columns represent the mean of all technical replicates with nucleotide levels ≥ LLOQ. Error bars of dashed columns indicate standard deviation of all technical replicates with nucleotide levels ≥ LLOQ. Dashed error bars indicate absence of a single technical replicate with measured nucleotide levels ≥ LLOQ. Values of dashed error bars represent the highest calculated value of all analyzed technical replicates with measured nucleotide levels < LLOQ. The exact nucleotide concentrations are summarized in Table 3 (Exp., exponential; Stat., stationary).

In a second step, we screened for di-nucleotide-based (putative) second messengers. The only di-nucleotide-based second messenger detected in any archaeon so far is 3′,5′-c-di-AMP. It was recently shown to be produced in the euryarchaeon *H. volcanii* by the corresponding di-adenylate cyclase DacZ and osmoregulation had been implicated as a major function of this nucleotide (Braun et al., 2019). Extraction of nucleotides from the cell extracts followed by LC-MS/MS confirmed the presence of 3′,5′-c-di-AMP during both the exponential and the stationary phase at concentrations similar to what was detected previously (Braun et al., 2019; Figure 3A). In contrast to *H. volcanii*, *S. acidocaldarius* cell extracts generated in this study did not contain any 3′,5′-c-di-AMP. A protein BLAST search using the sequences of an established bacterial (Rosenberg et al., 2015) and an archaeal (Braun et al., 2019) di-adenylate cyclases against the proteome of *S. acidocaldarius* yielded no significant hits. These observations are consistent with previous bioinformatical analyses showing that proteins containing the 3′,5′-c-di-AMP synthesizing diadenylate cyclase (DAC)-domain (COG1624) are absent in crenarchaeota, while being frequently found in euryarchaeota (Römling, 2008; Witte et al., 2008; He et al., 2020). This suggests that 3′,5′-c-di-AMP is likely to be used as second messenger by many euryarchaea, whereas crenarchaea do not seem to utilize this signaling nucleotide.

**TABLE 3:** Summary of measured and detected di-nucleotide-based (putative) second messengers.

Organism	*H. volcanii*		*S. acidocaldarius*	
Molecule	Levels during exponential growth [ng/mg protein]	Levels during stationary growth [ng/mg protein]	Levels during exponential growth [ng/mg protein]	Levels during stationary growth [ng/mg protein]
*3*′,*5*′*-c-di-GMP*	n.d.	n.d.	n.d.	n.d.
*3*′,*5*′*-c-di-AMP*	13.56 ± 1.34	15.02 ± 1.03	n.d.	n.d.
*5*′*-pGpG*	n.d.	n.d.	n.d.	n.d.
*5*′*-pApA*	≤ 0.92 ± 0.13	0.37 ± 0.04	n.d.	≥ 0
*2*′,*3*′*-cGAMP*	n.d.	n.d.	n.d.	n.d.
*3*′,*3*′*-cGAMP*	n.d.	n.d.	n.d.	n.d.
*3*′,*2*′*-cGAMP*	n.d.	n.d.	n.d.	n.d.
*Ap_4_A*	3.14 ± 1.88	0.48 ± 0.18	206.51 ± 26.61	13.40 ± 3.77

*(**±**, gives standard deviation; n.d., not detectable; ≤, average of all technical replicates ≥ LLOQ; ≥ 0, nucleotide detected but all technical replicates < LLOQ).*

With 3′,5′-c-di-AMP being present in *H. volcanii*, it was not surprising that its (intermediate) degradation product 5′-pApA, was also found in this euryarchaeon (Figure 3A). The concentration of 5′-pApA was, however, significantly lower than the concentration of 3′,5′-c-di-AMP. Especially samples from exponentially growing cultures contained only low amounts of 5′-pApA. Although all nine replicates of exponential cultures included 5′-pApA, only two of them contained sufficient amounts for a quantitative analysis. These very low levels of 5′-pApA suggests that most of this linear di-nucleotide in *H. volcanii* is further degraded to 5′-AMP, the final degradation product of 3′,5′-c-di-AMP (Commichau et al., 2019). The phosphodiesterases which are degrading 3′,5′-c-di-AMP and/or 5′-pApA in *H. volcanii* are currently unknown, as well as a potential function of 5′-pApA as second messenger. Samples from exponentially growing *S. acidocaldarius* cells did not exhibit any 5′-pApA. Yet, most samples (eight of nine replicates) from stationary *S. acidocaldarius* cultures included minor amounts of 5′-pApA (Figure 3B). However, as these amounts were in all samples below the quantification limit no valid average 5′-pApA level could be calculated. As *S. acidocaldarius* does not contain any 3′,5′-c-di-AMP these minor amounts of 5′-pApA do certainly not originate from the degradation of 3′,5′-c-di-AMP. As the genome of *S. acidocaldarius* has an A + T-content of 63% (Chen et al., 2005) it appears possible that the detected molecules of 5′-pApA are intermediate fragments from degraded genomic DNA.

Diadenosine tetraphosphate (Ap_4_A), which was shown in several bacteria to function as an stress induced second messenger (Lee et al., 1983; Pálfi et al., 1991; Kimura et al., 2017; Ferguson et al., 2020), could be detected in cell extracts from both archaeal model organisms (Figures 3A,B). For both species, levels of Ap_4_A were higher during exponential growth. However, samples from exponentially growing *H. volcanii* cells exhibited a quite broad fluctuation among individual technical replicates and among the biological replicates, with some samples even exhibiting a complete absence of Ap_4_A (Figure 3A). Intriguingly, Ap_4_A levels in exponentially growing *S. acidocaldarius* cells were the highest of all putative nucleotide-based second messengers detected in this study (Figure 3B). Noteworthy, exponential samples from *S. acidocaldarius* did not show a similar broad fluctuation for their Ap_4_A levels as observed for exponential samples from *H. volcanii*. The 16-fold difference between Ap_4_A levels in *S. acidocaldarius* cells during exponential growth and the stationary phase is the largest difference that was observed between the two phases within this study. Whether Ap_4_A is actually used in a second messenger context and what possible biological functions this di-nucleotide could have in *H. volcanii* and *S. acidocaldarius* is currently not known. The observed differences between exponential and stationary growth phases as well as the high amounts of Ap_4_A specifically produced by exponentially growing *S. acidocaldarius* cells imply a general physiological relevance of this di-nucleotide. Noteworthy, bioinformatical identification of any Ap_4_A synthesizing enzyme in *S. acidocaldarius* and *H. volcanii* is particularly complicated as a broad variety of aminoacyl-tRNA synthetases and also other enzymes like, for example, DNA and RNA ligases, are known to be capable of forming this di-nucleotide (Fraga and Fontes, 2011; Ferguson et al., 2020).

Interestingly, the very well established bacterial second messenger 3′,5′-c-di-GMP and its (intermediate) degradation product 5′-pGpG were not detected in the cell extracts of *H. volcanii* and *S. acidocaldarius*. This suggests that this cyclic di-nucleotide is, unlike to various bacteria, not a key regulatory molecule in these two species. Indeed, a protein BLAST search using the GGDEF-domain, the domain responsible for 3′,5′-c-di-GMP formation (Paul et al., 2004), of a di-guanylate cyclase from *E. coli* (Ryjenkov et al., 2005) against the genera *Haloferax* and *Sulfolobus* yielded no hits for the genus *Sulfolobus* and only three hits for the genus *Haloferax*, of which only one contained the complete GGDEF-motive (hypothetical protein; overall query cover: 40%, percent identity: 38.89%). This is in agreement with previous bioinformatical analyses which showed that not only proteins with a GGDEF-domain are almost completely absent in archaea, but also proteins with all other domains associated with 3′,5′-c-di-GMP signaling (e.g., EAL- or PilZ-domain) (Römling et al., 2013). Nevertheless, an analogous BLAST search against the entire domain of archaea yielded more than 300 hits of putative GGDEF-domain containing enzymes with many of them being predicted to belong to species of the recently discovered Asgard- and DPANN-superphyla. This suggests that 3′,5′-c-di-GMP might not entirely be absent in the archaeal domain of life.

In addition to 3′,5′-c-di-GMP and 5′-pGpG, cell extracts were also analyzed for the presence of the eukaryotic di-nucleotide-based second messengers 2′,3′-cGAMP and 3′,2′-cGAMP and their bacterial analog 3′,3′-cGAMP. None of these three isomers could be detected. The absence of 2′,3′-cGAMP in *H. volcanii* and *S. acidocaldarius* fits with the current idea of 2′,3′-cGAMP only being present in metazoa (Kranzusch et al., 2015). The recently discovered isomer 3′,2′-cGAMP was also not detected in any sample. As this isomer also contains an atypical 2-′5′ phosphodiester linkage it appears very likely that it is also only produced by metazoa. The fact that 3′,3′-cGAMP is absent in both species might suggest that both do not use any of the prokaryotic anti-phage defense mechanisms, which have been previously linked to bacterial 3′,3-′cGAMP production (Severin et al., 2018; Cohen et al., 2019). This idea is also supported by a complete lack of proteins in the genera of *Haloferax* and *Sulfolobus* sharing significant similarities with so far characterized bacterial cyclic GMP-AMP synthases (cGASs) (BLAST search using two characterized bacterial cGASs; Davies et al., 2012; Li et al., 2019). A look at the COGs for both, bacterial (ENOG5028K9C) and metazoan (KOG3963), cGASs unveils that each of them is specific for its respective domain and that they do not root in a common COG which would also include any archaeon.

### Cyclic Tetra-AMP Could Not Be Detected in *Haloferax volcanii* and *Sulfolobus acidocaldarius* Cell Extracts

Only a few cyclic oligo-nucleotide-based second messengers have been identified. An example of this is c-tetra-AMP (*n* = 4), which is known to occur, alongside with other isomers of cyclic oligo adenylate (*n* = 3–6) (cOA), in some crenarchaeal species, such as *Sa. solfataricus*, a species closely related to *S. acidocaldarius* (Rouillon et al., 2018). There, cOA was shown to be involved in type III CRISPR system mediated immunity (Kazlauskiene et al., 2017; Niewoehner et al., 2017; Rouillon et al., 2018). No c-tetra-AMP could be detected in any of the samples prepared for this study. As *S. acidocaldarius* encodes a functional type III CRISPR system that contains a Cas10 subunit (Zink et al., 2021) detection of c-tetra-AMP in extracts from this species could have been expected. However, as the cells in this study were not challenged with any invading virus it is plausible that the observed lack of c-tetra-AMP originates from the type III CRISPR system of *S. acidocaldarius* being inactive at the tested conditions. *H. volcanii* and other euryarchaea of the order Halobacteriales contain type I, but lack type III CRISPR systems (Maier et al., 2019), which certainly explains the absence of c-tetra-AMP (and thereby most likely the absence of cOA in general) in *H. volcanii*.

## Concluding Remark

The results of this study represent the first screening of cell extracts from an euryarchaeal and a crenarchaeal species for a multitude of currently known (and established) nucleotide-based second messengers using a modern and highly sensitive mass spectrometry method. It gives a comprehensive overview on a broad spectrum of (potential) small signaling molecules which are present in the archaeal model organisms *H. volcanii* and *S. acidocaldarius* under standard growth and experimental conditions. The function of second messengers includes rapid variations within their levels depending on changing environmental conditions. It thus appears reasonable that the here measured nucleotide levels might considerably change when different cultivation methods/circumstances are used. Even the appearance of a nucleotide species totally absent here (or vice versa) appears then possible. Determining the nucleotide levels in such differentiating growth experiments could help to elucidate the functions of several of the here reported (putative) second messengers in *H. volcanii* and *S. acidocaldarius*, and thereby also in archaea in more general.

## Data Availability Statement

The raw data supporting the conclusions of this article will be made available by the authors, upon request.

## Author Contributions

FB designed the experiments and analyzed data under supervision from S-VA. FB performed the growth of *H. volcanii* and the nucleotide extractions. AR performed the growth of *S. acidocaldarius*. HB and RS supervised LC-MS/MS nucleotide measurements. FB wrote the manuscript with input from HB, AR, RS, and S-VA. All authors contributed to the article and approved the submitted version.

## Conflict of Interest

The authors declare that the research was conducted in the absence of any commercial or financial relationships that could be construed as a potential conflict of interest.

## Publisher’s Note

All claims expressed in this article are solely those of the authors and do not necessarily represent those of their affiliated organizations, or those of the publisher, the editors and the reviewers. Any product that may be evaluated in this article, or claim that may be made by its manufacturer, is not guaranteed or endorsed by the publisher.

## References

[B1] Ablasser A. Goldeck M. Cavlar T. Deimling T. Witte G. Röhl I. (2013). cGAS produces a 2′-5′-linked cyclic dinucleotide second messenger that activates STING. *Nature* 498 380–384. 10.1038/nature12306 23722158 PMC4143541

[B2] Allers T. Ngo H.-P. Mevarech M. Lloyd R. G. (2004). Development of additional selectable markers for the halophilic archaeon Haloferax volcanii based on the leuB and trpA genes. *Appl. Environ. Microbiol.* 70 943–953. 10.1128/AEM.70.2.943-953.2004 14766575 PMC348920

[B3] An S.-Q. Chin K.-H. Febrer M. McCarthy Y. Yang J.-G. Liu C.-L. (2013). A cyclic GMP-dependent signalling pathway regulates bacterial phytopathogenesis. *EMBO J.* 32 2430–2438. 10.1038/emboj.2013.165 23881098 PMC3770947

[B4] Anderson T. R. (1982). Cyclic cytidine 3′,5′-monophosphate (cCMP) in cell regulation. *Mol. Cell. Endocrinol.* 28 373–385. 10.1016/0303-7207(82)90134-4 6295841

[B5] Atkinson G. C. Tenson T. Hauryliuk V. (2011). The RelA/SpoT homolog (RSH) superfamily: distribution and functional evolution of ppGpp synthetases and hydrolases across the tree of life. *PLoS One* 6:e23479. 10.1371/journal.pone.0023479 21858139 PMC3153485

[B6] Bähre H. Danker K. Y. Stasch J.-P. Kaever V. Seifert R. (2014). Nucleotidyl cyclase activity of soluble guanylyl cyclase in intact cells. *Biochem. Biophys. Res. Commun.* 443 1195–1199. 24380860 10.1016/j.bbrc.2013.12.108

[B7] Bähre H. Hartwig C. Munder A. Wolter S. Stelzer T. Schirmer B. (2015). cCMP and cUMP occur *in vivo*. *Biochem. Biophys. Res. Commun.* 460 909–914. 10.1016/j.bbrc.2015.03.115 25838203 PMC4765920

[B8] Bähre H. Kaever V. (2014). Measurement of 2′,3′-cyclic nucleotides by liquid chromatography-tandem mass spectrometry in cells. *J. Chromatogr. B Analyt. Technol. Biomed. Life Sci.* 964 208–211. 10.1016/j.jchromb.2014.02.046 24656940

[B9] Bähre H. Kaever V. (2017). Identification and Quantification of Cyclic Di-Guanosine Monophosphate and Its Linear Metabolites by Reversed-Phase LC-MS/MS. *Methods Mol. Biol.* 1657 45–58. 10.1007/978-1-4939-7240-1_5 28889285

[B10] Baltzinger M. Ebel J. P. Remy P. (1986). Accumulation of dinucleoside polyphosphates in Saccharomyces cerevisiae under stress conditions. High levels are associated with cell death. *Biochimie* 68 1231–1236. 10.1016/s0300-9084(86)80069-4 3098308

[B11] Barker J. R. Koestler B. J. Carpenter V. K. Burdette D. L. Waters C. M. Vance R. E. (2013). STING-dependent recognition of cyclic di-AMP mediates type I interferon responses during Chlamydia trachomatis infection. *mBio* 4 e00018–13. 10.1128/mBio.00018-13 23631912 PMC3663186

[B12] Bassler J. Schultz J. E. Lupas A. N. (2018). Adenylate cyclases: receivers, transducers, and generators of signals. *Cell. Signal.* 46 135–144. 10.1016/j.cellsig.2018.03.002 29563061

[B13] Battesti A. Bouveret E. (2006). Acyl carrier protein/SpoT interaction, the switch linking SpoT-dependent stress response to fatty acid metabolism. *Mol. Microbiol.* 62 1048–1063. 10.1111/j.1365-2958.2006.05442.x 17078815

[B14] Baumann A. Lange C. Soppa J. (2007). Transcriptome changes and cAMP oscillations in an archaeal cell cycle. *BMC Cell Biol.* 8:21. 10.1186/1471-2121-8-21 17562013 PMC1906763

[B15] Beauclerk A. A. Hummel H. Holmes D. J. Böck A. Cundliffe E. (1985). Studies of the GTPase domain of archaebacterial ribosomes. *Eur. J. Biochem.* 151 245–255. 10.1111/j.1432-1033.1985.tb09095.x 2411554

[B16] Beckert U. Wolter S. Hartwig C. Bähre H. Kaever V. Ladant D. (2014). ExoY from *Pseudomonas aeruginosa* is a nucleotidyl cyclase with preference for cGMP and cUMP formation. *Biochem. Biophys. Res. Commun.* 450 870–874. 10.1016/j.bbrc.2014.06.088 24971548 PMC4764875

[B17] Berthet J. Rall T. W. Sutherland E. W. (1957). The relationship of epinephrine and glucagon to liver phosphorylase. IV. Effect of epinephrine and glucagon on the reactivation of phosphorylase in liver homogenates. *J. Biol. Chem.* 224 463–475. 10.1016/s0021-9258(18)65045-8 13398422

[B18] Beste K. Y. Burhenne H. Kaever V. Stasch J.-P. Seifert R. (2012). Nucleotidyl cyclase activity of soluble guanylyl cyclase α1β1. *Biochemistry* 51 194–204. 10.1021/bi201259y 22122229

[B19] Beste K. Y. Seifert R. (2013). cCMP, cUMP, cTMP, cIMP and cXMP as possible second messengers: development of a hypothesis based on studies with soluble guanylyl cyclase α(1)β(1). *Biol. Chem.* 394 261–270. 10.1515/hsz-2012-0282 23087103

[B20] Blanco E. Fortunato S. Viggiano L. de Pinto M. C. (2020). Cyclic AMP: a Polyhedral Signalling Molecule in Plants. *Int. J. Mol. Sci.* 21:4862. 10.3390/ijms21144862 32660128 PMC7402341

[B21] Bloch A. Dutschman G. Maue R. (1974). Cytidine 3′,5′-monophosphate (cyclic CMP). II. Initiation of leukemia L-1210 cell growth *in vitro*. *Biochem. Biophys. Res. Commun.* 59 955–959. 10.1016/s0006-291x(74)80072-0 4369899

[B22] Bordeleau E. Oberc C. Ameen E. da Silva A. M. Yan H. (2014). Identification of cytidine 2′,3′-cyclic monophosphate and uridine 2′,3′-cyclic monophosphate in *Pseudomonas* fluorescens pfo-1 culture. *Bioorg. Med. Chem. Lett.* 24 4520–4522. 10.1016/j.bmcl.2014.07.080 25139571

[B23] Botsford J. L. Harman J. G. (1992). Cyclic AMP in prokaryotes. *Microbiol. Rev.* 56 100–122. 1315922 10.1128/mr.56.1.100-122.1992 PMC372856

[B24] Bowman L. Zeden M. S. Schuster C. F. Kaever V. Gründling A. (2016). New Insights into the Cyclic Di-adenosine Monophosphate (c-di-AMP) Degradation Pathway and the Requirement of the Cyclic Dinucleotide for Acid Stress Resistance in Staphylococcus aureus. *J. Biol. Chem.* 291 26970–26986. 10.1074/jbc.M116.747709 27834680 PMC5207132

[B25] Braun F. Thomalla L. Does C. Quax T. E. F. Allers T. Kaever V. (2019). Cyclic nucleotides in archaea: cyclic di-AMP in the archaeon Haloferax volcanii and its putative role. *Microbiologyopen* 8:e00829. 10.1002/mbo3.829 30884174 PMC6741144

[B26] Brevet A. Plateau P. Best-Belpomme M. Blanquet S. (1985). Variation of Ap4A and other dinucleoside polyphosphates in stressed Drosophila cells. *J. Biol. Chem.* 260 15566–15570. 10.1016/s0021-9258(17)36294-4 4066685

[B27] Brock T. D. Brock K. M. Belly R. T. Weiss R. L. (1972). Sulfolobus: a new genus of sulfur-oxidizing bacteria living at low pH and high temperature. *Arch. Mikrobiol.* 84 54–68. 10.1007/BF00408082 4559703

[B28] Burdette D. L. Monroe K. M. Sotelo-Troha K. Iwig J. S. Eckert B. Hyodo M. (2011). STING is a direct innate immune sensor of cyclic di-GMP. *Nature* 478 515–518. 10.1038/nature10429 21947006 PMC3203314

[B29] Burhenne H. Beste K. Spangler C. Voigt U. Kaever V. Seifert R. (2011). Determination of cytidine 3′,5′ -cyclic monophosphate and uridine 3′,5′ -cyclic monophosphate in mammalian cell systems and in human urine by high performance liquid chromatography/mass spectrometry. *Naunyn. Schmiedebergs. Arch. Pharmacol.* 383:P096.

[B30] Cadoret J.-C. Rousseau B. Perewoska I. Sicora C. Cheregi O. Vass I. (2005). Cyclic nucleotides, the photosynthetic apparatus and response to a UV-B stress in the Cyanobacterium Synechocystis sp. PCC 6803. *J. Biol. Chem.* 280 33935–33944. 10.1074/jbc.M503153200 16096278

[B31] Cashel M. Gallant J. (1969). Two compounds implicated in the function of the RC gene of *Escherichia coli*. *Nature* 221 838–841. 10.1038/221838a0 4885263

[B32] Cellini A. Scoarughi G. L. Poggiali P. Santino I. Sessa R. Donini P. (2004). Stringent control in the archaeal genus Sulfolobus. *Res. Microbiol.* 155 98–104. 10.1016/j.resmic.2003.11.006 14990261

[B33] Chen L. Brügger K. Skovgaard M. Redder P. She Q. Torarinsson E. (2005). The genome of Sulfolobus acidocaldarius, a model organism of the Crenarchaeota. *J. Bacteriol.* 187 4992–4999. 10.1128/jb.187.14.4992-4999.2005 15995215 PMC1169522

[B34] Chen Z. Schaap P. (2012). The prokaryote messenger c-di-GMP triggers stalk cell differentiation in Dictyostelium. *Nature* 488 680–683. 10.1038/nature11313 22864416 PMC3939355

[B35] Chen Z. Zhang X. Ying L. Dou D. Li Y. Bai Y. (2014). cIMP synthesized by sGC as a mediator of hypoxic contraction of coronary arteries. *Am. J. Physiol. Heart Circ. Physiol.* 307 328–336. 10.1152/ajpheart.00132.2014 24906916

[B36] Cimmino C. Scoarughi G. L. Donini P. (1993). Stringency and relaxation among the halobacteria. *J. Bacteriol.* 175 6659–6662. 10.1128/jb.175.20.6659-6662.1993 7691798 PMC206777

[B37] Clapham D. E. (2007). Calcium Signaling. *Cell* 131 1047–1058. 18083096 10.1016/j.cell.2007.11.028

[B38] Cohen D. Melamed S. Millman A. Shulman G. Oppenheimer-Shaanan Y. Kacen A. (2019). Cyclic GMP-AMP signalling protects bacteria against viral infection. *Nature* 574 691–695. 10.1038/s41586-019-1605-5 31533127

[B39] Commichau F. M. Heidemann J. L. Ficner R. Stülke J. (2019). Making and Breaking of an Essential Poison: the Cyclases and Phosphodiesterases That Produce and Degrade the Essential Second Messenger Cyclic di-AMP in Bacteria. *J. Bacteriol.* 201 e00462–18. 10.1128/JB.00462-18 30224435 PMC6287462

[B40] Corrigan R. M. Gründling A. (2013). Cyclic di-AMP: another second messenger enters the fray. *Nat. Rev. Microbiol.* 11 513–524. 10.1038/nrmicro3069 23812326

[B41] Coste H. Brevet A. Plateau P. Blanquet S. (1987). Non-adenylylated bis(5′-nucleosidyl) tetraphosphates occur in Saccharomyces cerevisiae and in *Escherichia coli* and accumulate upon temperature shift or exposure to cadmium. *J. Biol. Chem.* 262 12096–12103. 10.1016/s0021-9258(18)45321-5 3305502

[B42] Crasnier M. (1996). Cyclic AMP and catabolite repression. *Res. Microbiol.* 147 479–482. 10.1016/0923-2508(96)84002-2 9084758

[B43] Davies B. W. Bogard R. W. Young T. S. Mekalanos J. J. (2012). Coordinated regulation of accessory genetic elements produces cyclic di-nucleotides for V. cholerae virulence. *Cell* 149 358–370. 10.1016/j.cell.2012.01.053 22500802 PMC3620040

[B44] Desch M. Schinner E. Kees F. Hofmann F. Seifert R. Schlossmann J. (2010). Cyclic cytidine 3′,5′-monophosphate (cCMP) signals via cGMP kinase I. *FEBS Lett.* 584 3979–3984. 10.1016/j.febslet.2010.07.059 20691687

[B45] Diner E. J. Burdette D. L. Wilson S. C. Monroe K. M. Kellenberger C. A. Hyodo M. (2013). The innate immune DNA sensor cGAS produces a noncanonical cyclic dinucleotide that activates human STING. *Cell Rep.* 3 1355–1361. 10.1016/j.celrep.2013.05.009 23707065 PMC3706192

[B46] Dittmar F. Abdelilah-Seyfried S. Tschirner S. K. Kaever V. Seifert R. (2015). Temporal and organ-specific detection of cNMPs including cUMP in the zebrafish. *Biochem. Biophys. Res. Commun.* 468 708–712. 10.1016/j.bbrc.2015.11.020 26551461

[B47] Duggin I. G. Aylett C. H. S. Walsh J. C. Michie K. A. Wang Q. Turnbull L. (2015). CetZ tubulin-like proteins control archaeal cell shape. *Nature* 519 362–365. 10.1038/nature13983 25533961 PMC4369195

[B48] Escalante R. Wessels D. Soll D. R. Loomis W. F. (1997). Chemotaxis to cAMP and slug migration in Dictyostelium both depend on migA, a BTB protein. *Mol. Biol. Cell* 8 1763–1775. 10.1091/mbc.8.9.1763 9307972 PMC305735

[B49] Fahmi T. Port G. C. Cho K. H. (2017). c-di-AMP: an Essential Molecule in the Signaling Pathways that Regulate the Viability and Virulence of Gram-Positive Bacteria. *Genes* 8:197. 10.3390/genes8080197 28783096 PMC5575661

[B50] Ferguson F. McLennan A. G. Urbaniak M. D. Jones N. J. Copeland N. A. (2020). Re-evaluation of Diadenosine Tetraphosphate (Ap4A) From a Stress Metabolite to Bona Fide Secondary Messenger. *Front. Mol. Biosci.* 7:606807. 10.3389/fmolb.2020.606807 33282915 PMC7705103

[B51] Flärdh K. Axberg T. Albertson N. H. Kjelleberg S. (1994). Stringent control during carbon starvation of marine Vibrio sp. strain S14: molecular cloning, nucleotide sequence, and deletion of the relA gene. *J. Bacteriol.* 176 5949–5957. 10.1128/jb.176.19.5949-5957.1994 7928955 PMC196811

[B52] Flodgaard H. Klenow H. (1982). Abundant amounts of diadenosine 5′,5”′-P1,P4-tetraphosphate are present and releasable, but metabolically inactive, in human platelets. *Biochem. J.* 208 737–742. 10.1042/bj2080737 6299279 PMC1154025

[B53] Fontaine B. M. Martin K. S. Garcia-Rodriguez J. M. Jung C. Briggs L. Southwell J. E. (2018). RNase I regulates *Escherichia coli* 2′,3′-cyclic nucleotide monophosphate levels and biofilm formation. *Biochem. J.* 475 1491–1506. 10.1042/BCJ20170906 29555843 PMC6452634

[B54] Fraga H. Fontes R. (2011). Enzymatic synthesis of mono and dinucleoside polyphosphates. *Biochim. Biophys. Acta* 1810 1195–1204. 10.1016/j.bbagen.2011.09.010 21978831

[B55] Gallant J. Palmer L. Pao C. C. (1977). Anomalous synthesis of ppGpp in growing cells. *Cell* 11 181–185. 10.1016/0092-8674(77)90329-4 326415

[B56] Gancedo J. M. (2013). Biological roles of cAMP: variations on a theme in the different kingdoms of life. *Biol. Rev.* 88 645–668. 10.1111/brv.12020 23356492

[B57] Gao P. Ascano M. Wu Y. Barchet W. Gaffney B. L. Zillinger T. (2013). Cyclic [G(2′,5′)pA(3′,5′)p] is the metazoan second messenger produced by DNA-activated cyclic GMP-AMP synthase. *Cell* 153 1094–1107. 10.1016/j.cell.2013.04.046 23647843 PMC4382009

[B58] Garrison P. N. Barnes L. D. (1984). Assay of adenosine 5′-P1-tetraphospho-P4-5”′-adenosine and adenosine 5′-P1-tetraphospho-P4-5”′-guanosine in Physarum polycephalum and other eukaryotes. An isocratic high-pressure liquid-chromatography method. *Biochem. J.* 217 805–811. 10.1042/bj2170805 6370234 PMC1153284

[B59] Garrison P. N. Mathis S. A. Barnes L. D. (1989). Changes in diadenosine tetraphosphate levels in Physarum polycephalum with different oxygen concentrations. *J. Bacteriol.* 171 1506–1512. 10.1128/jb.171.3.1506-1512.1989 2921243 PMC209773

[B60] Genschik P. Billy E. Swianiewicz M. Filipowicz W. (1997). The human RNA 3′-terminal phosphate cyclase is a member of a new family of proteins conserved in Eucarya, Bacteria and Archaea. *EMBO J.* 16 2955–2967. 10.1093/emboj/16.10.2955 9184239 PMC1169903

[B61] Goldman S. R. Sharp J. S. Vvedenskaya I. O. Livny J. Dove S. L. Nickels B. E. (2011). NanoRNAs prime transcription initiation *in vivo*. *Mol. Cell* 42 817–825. 10.1016/j.molcel.2011.06.005 21700226 PMC3130991

[B62] Göttle M. Dove S. Kees F. Schlossmann J. Geduhn J. König B. (2010). Cytidylyl and uridylyl cyclase activity of Bacillus anthracis edema factor and Bordetella pertussis CyaA. *Biochemistry* 49 5494–5503. 10.1021/bi100684g 20521845 PMC2951761

[B63] Hartwig C. Bähre H. Wolter S. Beckert U. Kaever V. Seifert R. (2014). cAMP, cGMP, cCMP and cUMP concentrations across the tree of life: high cCMP and cUMP levels in astrocytes. *Neurosci. Lett.* 579 183–187. 10.1016/j.neulet.2014.07.019 25062586

[B64] He J. Yin W. Galperin M. Y. Chou S.-H. (2020). Cyclic di-AMP, a second messenger of primary importance: tertiary structures and binding mechanisms. *Nucleic Acids Res.* 48 2807–2829. 10.1093/nar/gkaa112 32095817 PMC7102992

[B65] Hilgemann D. W. Dai G. Collins A. Lariccia V. Magi S. Deisl C. (2018). Lipid signaling to membrane proteins: from second messengers to membrane domains and adapter-free endocytosis. *J. Gen. Physiol.* 150 211–224. 10.1085/jgp.201711875 29326133 PMC5806671

[B66] Holleufer A. Winther K. G. Gad H. H. Ai X. Chen Y. Li L. (2021). Two cGAS-like receptors induce antiviral immunity in Drosophila. *Nature* 597 114–118. 10.1038/s41586-021-03800-z 34261128

[B67] Hood R. D. Higgins S. A. Flamholz A. Nichols R. J. Savage D. F. (2016). The stringent response regulates adaptation to darkness in the cyanobacterium Synechococcus elongatus. *Proc. Natl. Acad. Sci. U. S. A.* 113 E4867–E4876. 10.1073/pnas.1524915113 27486247 PMC4995992

[B68] Ihara Y. Ohta H. Masuda S. (2015). A highly sensitive quantification method for the accumulation of alarmone ppGpp in Arabidopsis thaliana using UPLC-ESI-qMS/MS. *J. Plant Res.* 128 511–518. 10.1007/s10265-015-0711-1 25752614

[B69] Ito D. Kawamura H. Oikawa A. Ihara Y. Shibata T. Nakamura N. (2020). ppGpp functions as an alarmone in metazoa. *Commun. Biol.* 3:671. 10.1038/s42003-020-01368-4 PMC7666150 33188280

[B70] Jackson E. K. (2011). The 2′,3′-cAMP-adenosine pathway. *Am. J. Physiol. Renal Physiol.* 301 F1160–F1167. 21937608 10.1152/ajprenal.00450.2011 PMC3233866

[B71] Jackson E. K. (2017). Discovery and Roles of 2′,3′-cAMP in Biological Systems. *Handb. Exp. Pharmacol.* 238 229–252. 10.1007/164_2015_40 26721674

[B72] Jia X. Fontaine B. M. Strobel F. Weinert E. E. (2014). A facile and sensitive method for quantification of cyclic nucleotide monophosphates in mammalian organs: basal levels of eight cNMPs and identification of 2′,3′-cIMP. *Biomolecules* 4 1070–1092. 10.3390/biom4041070 25513747 PMC4279170

[B73] Johnson J.-L. F. Leroux M. R. (2010). cAMP and cGMP signaling: sensory systems with prokaryotic roots adopted by eukaryotic cilia. *Trends Cell Biol.* 20 435–444. 10.1016/j.tcb.2010.05.005 20541938

[B74] Kang Y. Liu R. Wu J.-X. Chen L. (2019). Structural insights into the mechanism of human soluble guanylate cyclase. *Nature* 574 206–210. 10.1038/s41586-019-1584-6 31514202

[B75] Kazlauskiene M. Kostiuk G. Venclovas È Tamulaitis G. Siksnys V. (2017). A cyclic oligonucleotide signaling pathway in type III CRISPR-Cas systems. *Science* 357 605–609. 10.1126/science.aao0100 28663439

[B76] Khannpnavar B. Mehta V. Qi C. Korkhov V. (2020). Structure and function of adenylyl cyclases, key enzymes in cellular signaling. *Curr. Opin. Struct. Biol.* 63 34–41. 10.1016/j.sbi.2020.03.003 32334344

[B77] Kimura Y. Tanaka C. Sasaki K. Sasaki M. (2017). High concentrations of intracellular Ap4A and/or Ap5A in developing Myxococcus xanthus cells inhibit sporulation. *Microbiology* 163 86–93. 10.1099/mic.0.000403 27902428

[B78] Koonin E. V. Makarova K. S. (2018). Discovery of Oligonucleotide Signaling Mediated by CRISPR-Associated Polymerases Solves Two Puzzles but Leaves an Enigma. *ACS Chem. Biol.* 13 309–312. 10.1021/acschembio.7b00713 28937734 PMC11075118

[B79] Kranzusch P. J. (2019). cGAS and CD-NTase enzymes: structure, mechanism, and evolution. *Curr. Opin. Struct. Biol.* 59 178–187. 10.1016/j.sbi.2019.08.003 31593902 PMC7127440

[B80] Kranzusch P. J. Wilson S. C. Lee A. S. Y. Berger J. M. Doudna J. A. Vance R. E. (2015). Ancient Origin of cGAS-STING Reveals Mechanism of Universal 2′,3′ cGAMP Signaling. *Mol. Cell* 59 891–903. 10.1016/j.molcel.2015.07.022 26300263 PMC4575873

[B81] Kuipers K. Gallay C. Martínek V. Rohde M. Martínková M. van der Beek S. L. (2016). Highly conserved nucleotide phosphatase essential for membrane lipid homeostasis in Streptococcus pneumoniae. *Mol. Microbiol.* 101 12–26. 10.1111/mmi.13312 26691161

[B82] Kuwayama H. Ecke M. Gerisch G. Van Haastert P. J. (1996). Protection against osmotic stress by cGMP-mediated myosin phosphorylation. *Science* 271 207–209. 10.1126/science.271.5246.207 8539621

[B83] Lazzarini R. A. Cashel M. Gallant J. (1971). On the regulation of guanosine tetraphosphate levels in stringent and relaxed strains of *Escherichia coli*. *J. Biol. Chem.* 246 4381–4385. 10.1016/s0021-9258(18)62023-x 4937124

[B84] Lee P. C. Bochner B. R. Ames B. N. (1983). AppppA, heat-shock stress, and cell oxidation. *Proc. Natl. Acad. Sci. U. S. A.* 80 7496–7500. 10.1073/pnas.80.24.7496 6369319 PMC389978

[B85] Lee Y.-N. Nechushtan H. Figov N. Razin E. (2004). The function of lysyl-tRNA synthetase and Ap4A as signaling regulators of MITF activity in FcepsilonRI-activated mast cells. *Immunity* 20 145–151. 10.1016/s1074-7613(04)00020-2 14975237

[B86] Leichtling B. H. Rickenberg H. V. Seely R. J. Fahrney D. E. Pace N. R. (1986). The occurrence of cyclic AMP in archaebacteria. *Biochem. Biophys. Res. Commun.* 136 1078–1082. 10.1016/0006-291x(86)90443-2 3013165

[B87] Leung S. W. S. Gao Y. Vanhoutte P. M. (2015). “3′,5′-cIMP as Potential Second Messenger in the Vascular Wall,” in *Non-canonical Cyclic Nucleotides*, ed. Seifert R. (Cham: Springer International Publishing), 209–228. 10.1007/164_2015_39

[B88] Li F. Cimdins A. Rohde M. Jänsch L. Kaever V. Nimtz M. (2019). DncV Synthesizes Cyclic GMP-AMP and Regulates Biofilm Formation and Motility in *Escherichia coli* ECOR31. *mBio* 10 e02492–18. 10.1128/mBio.02492-18 30837338 PMC6401482

[B89] Liscovitch M. Cantley L. C. (1994). Lipid second messengers. *Cell* 77 329–334. 10.1016/0092-8674(94)90148-1 8181054

[B90] Liu C. Sun D. Zhu J. Liu J. Liu W. (2020). The Regulation of Bacterial Biofilm Formation by cAMP-CRP: a Mini-Review. *Front. Microbiol.* 11:802. 10.3389/fmicb.2020.00802 32528421 PMC7247823

[B91] Magnusson L. U. Farewell A. Nyström T. (2005). ppGpp: a global regulator in *Escherichia coli*. *Trends Microbiol.* 13 236–242. 10.1016/j.tim.2005.03.008 15866041

[B92] Maiellaro I. Lohse M. J. Kittel R. J. Calebiro D. (2016). cAMP Signals in Drosophila Motor Neurons Are Confined to Single Synaptic Boutons. *Cell Rep.* 17 1238–1246. 10.1016/j.celrep.2016.09.090 27783939 PMC5098120

[B93] Maier L.-K. Stachler A.-E. Brendel J. Stoll B. Fischer S. Haas K. A. (2019). The nuts and bolts of the Haloferax CRISPR-Cas system I-B. *RNA Biol.* 16 469–480. 10.1080/15476286.2018.1460994 29649958 PMC6546412

[B94] Marden J. N. Dong Q. Roychowdhury S. Berleman J. E. Bauer C. E. (2011). Cyclic GMP controls Rhodospirillum centenum cyst development. *Mol. Microbiol.* 79 600–615. 10.1111/j.1365-2958.2010.07513.x 21214648 PMC4273943

[B95] Marx J. L. (1972). Cyclic AMP in Brain: role in Synaptic Transmission. *Science* 178 1188–1190. 10.1126/science.178.4066.1188 17748980

[B96] McDonough K. A. Rodriguez A. (2011). The myriad roles of cyclic AMP in microbial pathogens: from signal to sword. *Nat. Rev. Microbiol.* 10 27–38. 10.1038/nrmicro2688 22080930 PMC3785115

[B97] McLennan A. G. Prescott M. (1984). Diadenosine 5′,5′”-P1,P4-tetraphosphate in developing embryos of Artemia. *Nucleic Acids Res.* 12 1609–1619. 10.1093/nar/12.3.1609 6701090 PMC318602

[B98] Miras-Portugal M. T. Pintor J. Gualix J. (2003). Ca2+ signalling in brain synaptosomes activated by dinucleotides. *J. Membr. Biol.* 194 1–10. 10.1007/s00232-003-2024-x 14502438

[B99] Monds R. D. Newell P. D. Wagner J. C. Schwartzman J. A. Lu W. Rabinowitz J. D. (2010). Di-adenosine tetraphosphate (Ap4A) metabolism impacts biofilm formation by *Pseudomonas* fluorescens via modulation of c-di-GMP-dependent pathways. *J. Bacteriol.* 192 3011–3023. 10.1128/JB.01571-09 20154123 PMC2901679

[B100] Nan Y. Zeng X. Jin Z. Li N. Chen Z. Chen J. (2020). PDE1 or PDE5 inhibition augments NO-dependent hypoxic constriction of porcine coronary artery via elevating inosine 3′,5′-cyclic monophosphate level. *J. Cell. Mol. Med.* 24 14514–14524. 10.1111/jcmm.16078 33169529 PMC7754025

[B101] Newton A. C. Bootman M. D. Scott J. D. (2016). Second Messengers. *Cold Spring Harb. Perspect. Biol.* 8:a005926. 10.1101/cshperspect.a005926 PMC4968160 27481708

[B102] Newton R. P. Kingston E. E. Overton A. (1998). Mass spectrometric identification of cyclic nucleotides released by the bacterium Corynebacterium murisepticum into the culture medium. *Rapid Commun. Mass Spectrom.* 12 729–735. 10.1002/(sici)1097-0231(19980615)12:11<729::aid-rcm217>3.0.co;2-b

[B103] Nickels B. E. Dove S. L. (2011). NanoRNAs: a class of small RNAs that can prime transcription initiation in bacteria. *J. Mol. Biol.* 412 772–781. 10.1016/j.jmb.2011.06.015 21704045 PMC3184357

[B104] Niewoehner O. Garcia-Doval C. Rostøl J. T. Berk C. Schwede F. Bigler L. (2017). Type III CRISPR-Cas systems produce cyclic oligoadenylate second messengers. *Nature* 548 543–548. 10.1038/nature23467 28722012

[B105] Nisbett L.-M. Boon E. M. (2016). Nitric Oxide Regulation of H-NOX Signaling Pathways in Bacteria. *Biochemistry* 55 4873–4884. 10.1021/acs.biochem.6b00754 27479081 PMC5152592

[B106] Nishimura A. Moriya S. Ukai H. Nagai K. Wachi M. Yamada Y. (1997). Diadenosine 5′,5″′-P1,P4-tetraphosphate (Ap4A) controls the timing of cell division in *Escherichia coli*. *Genes Cells* 2 401–413. 10.1046/j.1365-2443.1997.1300328.x 9286857

[B107] Pabst M. Grass J. Fischl R. Léonard R. Jin C. Hinterkörner G. (2010). Nucleotide and nucleotide sugar analysis by liquid chromatography-electrospray ionization-mass spectrometry on surface-conditioned porous graphitic carbon. *Anal. Chem.* 82 9782–9788. 10.1021/ac101975k 21043458 PMC2995335

[B108] Pálfi Z. Surányi G. Borbély G. (1991). Alterations in the accumulation of adenylylated nucleotides in heavy-metal-ion-stressed and heat-stressed Synechococcus sp. strain PCC 6301, a cyanobacterium, in light and dark. *Biochem. J.* 276 487–491. 10.1042/bj2760487 1904720 PMC1151117

[B109] Paul R. Weiser S. Amiot N. C. Chan C. Schirmer T. Giese B. (2004). Cell cycle-dependent dynamic localization of a bacterial response regulator with a novel di-guanylate cyclase output domain. *Genes Dev.* 18 715–727. 10.1101/gad.289504 15075296 PMC387245

[B110] Plesner P. Ottesen M. (1980). Determination of diadenosine tetraphosphate in biological material by high pressure liquid chromatography. *Carlsberg Res. Commun.* 45 1–8. 10.1016/0003-2697(83)90313-5

[B111] Rall T. W. Sutherland E. W. (1958). Formation of a cyclic adenine ribonucleotide by tissue particles. *J. Biol. Chem.* 232 1065–1076. 10.1016/s0021-9258(19)77422-5 13549487

[B112] Rao F. See R. Y. Zhang D. Toh D. C. Ji Q. Liang Z.-X. (2010). YybT Is a Signaling Protein That Contains a Cyclic Dinucleotide Phosphodiesterase Domain and a GGDEF Domain with ATPase Activity. *J. Biol. Chem.* 285 473–482. 10.1074/jbc.M109.040238 19901023 PMC2804195

[B113] Ren J. Mi Z. Stewart N. A. Jackson E. K. (2009). Identification and quantification of 2′,3′-cAMP release by the kidney. *J. Pharmacol. Exp. Ther.* 328 855–865. 10.1124/jpet.108.146712 19033554 PMC2646794

[B114] Römling U. (2008). Great times for small molecules: c-di-AMP, a second messenger candidate in Bacteria and Archaea. *Sci. Signal.* 1:e39. 10.1126/scisignal.133pe39 18714086

[B115] Römling U. Galperin M. Y. Gomelsky M. (2013). Cyclic di-GMP: the first 25 years of a universal bacterial second messenger. *Microbiol. Mol. Biol. Rev.* 77 1–52. 10.1128/MMBR.00043-12 23471616 PMC3591986

[B116] Römling U. Gomelsky M. Galperin M. Y. (2005). C-di-GMP: the dawning of a novel bacterial signalling system. *Mol. Microbiol.* 57 629–639. 10.1111/j.1365-2958.2005.04697.x 16045609

[B117] Rosenberg J. Dickmanns A. Neumann P. Gunka K. Arens J. Kaever V. (2015). Structural and biochemical analysis of the essential diadenylate cyclase CdaA from Listeria monocytogenes. *J. Biol. Chem.* 290 6596–6606. 10.1074/jbc.M114.630418 25605729 PMC4358292

[B118] Rouillon C. Athukoralage J. S. Graham S. Grüschow S. White M. F. (2018). Control of cyclic oligoadenylate synthesis in a type III CRISPR system. *Elife* 7:e36734. 10.7554/eLife.36734 29963983 PMC6053304

[B119] Rybalkin S. D. Yan C. Bornfeldt K. E. Beavo J. A. (2003). Cyclic GMP phosphodiesterases and regulation of smooth muscle function. *Circ. Res.* 93 280–291. 10.1161/01.res.0000087541.15600.2b 12933699

[B120] Ryjenkov D. A. Tarutina M. Moskvin O. V. Gomelsky M. (2005). Cyclic diguanylate is a ubiquitous signaling molecule in bacteria: insights into biochemistry of the GGDEF protein domain. *J. Bacteriol.* 187 1792–1798. 10.1128/JB.187.5.1792-1798.2005 15716451 PMC1064016

[B121] Schäfer H. Beckert B. Frese C. K. Steinchen W. Nuss A. M. Beckstette M. (2020). The alarmones (p)ppGpp are part of the heat shock response of Bacillus subtilis. *PLoS Genet.* 16:e1008275. 10.1371/journal.pgen.1008275 32176689 PMC7098656

[B122] Scoarughi G. L. Cimmino C. Donini P. (1995). Lack of production of (p)ppGpp in Halobacterium volcanii under conditions that are effective in the eubacteria. *J. Bacteriol.* 177 82–85. 10.1128/jb.177.1.82-85.1995 7798153 PMC176559

[B123] Seifert R. (2015). cCMP and cUMP: emerging second messengers. *Trends Biochem. Sci.* 40 8–15. 10.1016/j.tibs.2014.10.008 25435399

[B124] Seifert R. (2017). “cCMP and cUMP Across the Tree of Life: From cCMP and cUMP Generators to cCMP- and cUMP-Regulated Cell Functions,” in *Non-canonical Cyclic Nucleotides*, ed. Seifert R. (Cham: Springer International Publishing), 3–23. 10.1007/164_2016_5005 28181008

[B125] Severin G. B. Ramliden M. S. Hawver L. A. Wang K. Pell M. E. Kieninger A.-K. (2018). Direct activation of a phospholipase by cyclic GMP-AMP in El Tor *Vibrio cholerae*. *Proc. Natl. Acad. Sci. U. S. A.* 115 E6048–E6055. 10.1073/pnas.1801233115 29891656 PMC6042076

[B126] Shemarova I. V. (2009). cAMP-dependent signal pathways in unicellular eukaryotes. *Crit. Rev. Microbiol.* 35 23–42. 10.1080/10408410802645646 19514907

[B127] Shigematsu M. Kawamura T. Kirino Y. (2018). Generation of 2′,3′-Cyclic Phosphate-Containing RNAs as a Hidden Layer of the Transcriptome. *Front. Genet.* 9:562. 10.3389/fgene.2018.00562 30538719 PMC6277466

[B128] Slavik K. M. Morehouse B. R. Ragucci A. E. Zhou W. Ai X. Chen Y. (2021). cGAS-like receptors sense RNA and control 3′2′-cGAMP signaling in Drosophila. *Nature* 597 109–113. 10.1038/s41586-021-03743-5 34261127 PMC8410604

[B129] Smith K. D. Lipchock S. V. Strobel S. A. (2012). Structural and biochemical characterization of linear dinucleotide analogues bound to the c-di-GMP-I aptamer. *Biochemistry* 51 425–432. 10.1021/bi2016662 22148472 PMC3257324

[B130] Spangler C. Böhm A. Jenal U. Seifert R. Kaever V. (2010). A liquid chromatography-coupled tandem mass spectrometry method for quantitation of cyclic di-guanosine monophosphate. *J. Microbiol. Methods* 81 226–231. 10.1016/j.mimet.2010.03.020 20385176

[B131] Spira B. Silberstein N. Yagil E. (1995). Guanosine 3′,5′-bispyrophosphate (ppGpp) synthesis in cells of *Escherichia coli* starved for Pi. *J. Bacteriol.* 177 4053–4058. 10.1128/jb.177.14.4053-4058.1995 7608079 PMC177136

[B132] Stelitano V. Giardina G. Paiardini A. Castiglione N. Cutruzzolà F. Rinaldo S. (2013). C-di-GMP hydrolysis by *Pseudomonas aeruginosa* HD-GYP phosphodiesterases: analysis of the reaction mechanism and novel roles for pGpG. *PLoS One* 8:e74920. 10.1371/journal.pone.0074920 24066157 PMC3774798

[B133] Stryer L. (1986). Cyclic GMP cascade of vision. *Annu. Rev. Neurosci.* 9 87–119. 10.1146/annurev.ne.09.030186.000511 2423011

[B134] Stülke J. Krüger L. (2020). Cyclic di-AMP Signaling in Bacteria. *Annu. Rev. Microbiol.* 74 159–179. 10.1146/annurev-micro-020518-115943 32603625

[B135] Sutherland E. W. Wosilait W. D. (1956). The relationship of epinephrine and glucagon to liver phosphorylase. I. Liver phosphorylase; preparation and properties. *J. Biol. Chem.* 218 459–468. 10.1016/s0021-9258(18)65909-5 13278353

[B136] Tal N. Morehouse B. R. Millman A. Stokar-Avihail A. Avraham C. Fedorenko T. (2021). Cyclic CMP and cyclic UMP mediate bacterial immunity against phages. *Cell.* [Online ahead of print],. 10.1016/j.cell.2021.09.031 34644530 PMC9070634

[B137] Tarusawa T. Ito S. Goto S. Ushida C. Muto A. Himeno H. (2016). (p)ppGpp-dependent and -independent pathways for salt tolerance in *Escherichia coli*. *J. Biochem.* 160 19–26. 10.1093/jb/mvw008 26823481

[B138] Thompson J. E. Venegas F. D. Raines R. T. (1994). Energetics of catalysis by ribonucleases: fate of the 2′,3′-cyclic phosphodiester intermediate. *Biochemistry* 33 7408–7414. 10.1021/bi00189a047 8003506

[B139] Tozawa Y. Nomura Y. (2011). Signalling by the global regulatory molecule ppGpp in bacteria and chloroplasts of land plants. *Plant Biol.* 13 699–709. 10.1111/j.1438-8677.2011.00484.x 21815973

[B140] Uematsu K. Fukui Y. (2008). Role and regulation of cAMP in seed germination of Phacelia tanacetifolia. *Plant Physiol. Biochem.* 46 768–774. 10.1016/j.plaphy.2007.10.015 18657429

[B141] Vahlensieck U. Bokník P. Gombosová I. Huke S. Knapp J. Linck B. (1999). Inotropic effects of diadenosine tetraphosphate (AP4A) in human and animal cardiac preparations. *J. Pharmacol. Exp. Ther.* 288 805–813. 9918592

[B142] Valentini M. Filloux A. (2016). Biofilms and Cyclic di-GMP (c-di-GMP) Signaling: lessons from *Pseudomonas aeruginosa* and Other Bacteria. *J. Biol. Chem.* 291 12547–12555. 10.1074/jbc.R115.711507 27129226 PMC4933438

[B143] Valentini M. Filloux A. (2019). Multiple Roles of c-di-GMP Signaling in Bacterial Pathogenesis. *Annu. Rev. Microbiol.* 73 387–406. 10.1146/annurev-micro-020518-115555 31500536

[B144] Van Damme T. Blancquaert D. Couturon P. Van Der Straeten D. Sandra P. Lynen F. (2014). Wounding stress causes rapid increase in concentration of the naturally occurring 2′,3′-isomers of cyclic guanosine- and cyclic adenosine monophosphate (cGMP and cAMP) in plant tissues. *Phytochemistry* 103 59–66. 10.1016/j.phytochem.2014.03.013 24735826

[B145] Vinella D. Albrecht C. Cashel M. D’Ari R. (2005). Iron limitation induces SpoT-dependent accumulation of ppGpp in *Escherichia coli*. *Mol. Microbiol.* 56 958–970. 10.1111/j.1365-2958.2005.04601.x 15853883

[B146] Vogt M. S. Ngouoko Nguepbeu R. R. Mohr M. K. F. Albers S.-V. Essen L.-O. Banerjee A. (2021). The archaeal triphosphate tunnel metalloenzyme SaTTM defines structural determinants for the diverse activities in the CYTH protein family. *J. Biol. Chem.* 297:100820. 10.1016/j.jbc.2021.100820 34029589 PMC8233210

[B147] Whiteley A. T. Eaglesham J. B. de Oliveira Mann C. C. Morehouse B. R. Lowey B. Nieminen E. A. (2019). Bacterial cGAS-like enzymes synthesize diverse nucleotide signals. *Nature* 567 194–199. 10.1038/s41586-019-0953-5 30787435 PMC6544370

[B148] Witte G. Hartung S. Büttner K. Hopfner K.-P. (2008). Structural biochemistry of a bacterial checkpoint protein reveals diadenylate cyclase activity regulated by DNA recombination intermediates. *Mol. Cell* 30 167–178. 10.1016/j.molcel.2008.02.020 18439896

[B149] Wolfe A. J. Visick K. L. (2008). Get the message out: cyclic-Di-GMP regulates multiple levels of flagellum-based motility. *J. Bacteriol.* 190 463–475. 10.1128/JB.01418-07 17993515 PMC2223684

[B150] Wolter S. Golombek M. Seifert R. (2011). Differential activation of cAMP- and cGMP-dependent protein kinases by cyclic purine and pyrimidine nucleotides. *Biochem. Biophys. Res. Commun.* 415 563–566. 10.1016/j.bbrc.2011.10.093 22074826

[B151] Wu J. Sun L. Chen X. Du F. Shi H. Chen C. (2013). Cyclic GMP-AMP is an endogenous second messenger in innate immune signaling by cytosolic DNA. *Science* 339 826–830. 10.1126/science.1229963 23258412 PMC3855410

[B152] Zhang X. Shi H. Wu J. Zhang X. Sun L. Chen C. (2013). Cyclic GMP-AMP containing mixed phosphodiester linkages is an endogenous high-affinity ligand for STING. *Mol. Cell* 51 226–235. 10.1016/j.molcel.2013.05.022 23747010 PMC3808999

[B153] Zhou L. Zhu D.-Y. (2009). Neuronal nitric oxide synthase: structure, subcellular localization, regulation, and clinical implications. *Nitric oxide Biol. Chem.* 20 223–230. 10.1016/j.niox.2009.03.001 19298861

[B154] Zink I. A. Fouqueau T. Tarrason Risa G. Werner F. Baum B. Bläsi U. (2021). Comparative CRISPR type III-based knockdown of essential genes in hyperthermophilic Sulfolobales and the evasion of lethal gene silencing. *RNA Biol.* 18 421–434. 10.1080/15476286.2020.1813411 32957821 PMC7951960

[B155] Zong X. Krause S. Chen C.-C. Krüger J. Gruner C. Cao-Ehlker X. (2012). Regulation of Hyperpolarization-activated Cyclic Nucleotide-gated (HCN) Channel Activity by cCMP. *J. Biol. Chem.* 287 26506–26512. 22715094 10.1074/jbc.M112.357129 PMC3410992

