# Supplementary material for: Putative Nucleotide-Based Second Messengers in the Archaeal Model Organisms Haloferax volcanii and Sulfolobus acidocaldarius
Source: Front Microbiol. 2021 Nov 22;12:779012. doi: 10.3389/fmicb.2021.779012 (PMC8646023; doi:10.3389/fmicb.2021.779012)
Supplement: Supplementary file 1 [file Data_Sheet_1.PDF]

Supplementary information

Putative nucleotide-based second messengers in the archaeal model organisms *Haloferax volcanii* and *Sulfolobus acidocaldarius*

Frank Braun, Alejandra Recalde, Heike Bähre, ,Roland Seifert, Sonja-Verena Albers

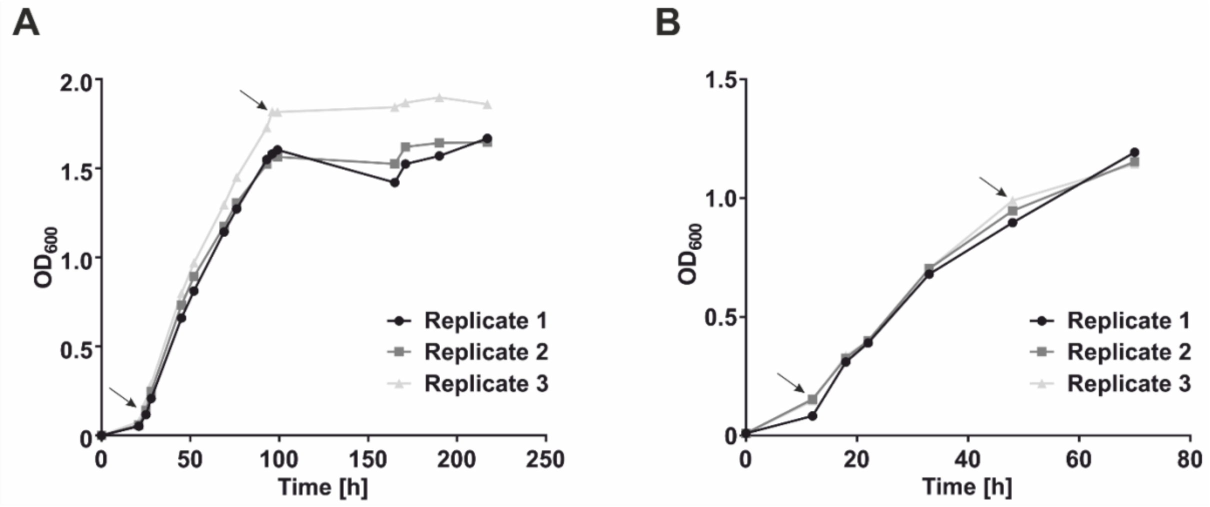

Fig. S1: Growth curves of **(A)** *H. volcanii* wild type (H26) in CAB medium and **(B)** *S. acidocaldarius* wild type (MW001) in Brock medium. Each line represents the mean of an individual biological replicate. Each biological replicate consists out of three technical replicates. Arrows indicate time points of cell material sampling for nucleotide extraction.

Table S1: Strains used in this study:

Strain name	Genotype	Reference/Source
H. volcanii		
H26	Δ*pyrE2*	(1)
S. acidocaldarius		
MW001	Δ*pyrEF*	(2)

Table S2: Summary of levels of measured and detected mono-, di-, and oligo-nucleotide-based (putative) second messengers in pmol/mg protein:

**Organism**	***H. volcanii***		***S. acidocaldarius***	
**Molecule**	**Levels during exponential growth [pmol/mg protein]**	**Levels during stationary growth [pmol/mg protein]**	**Levels during exponential growth [pmol/mg protein]**	**Levels during stationary growth [pmol/mg protein]**
**3’,5’-cAMP**	6.50 ± 0.58	2.11 ± 0.79	1.12 ± 0.22	0.18 ± 0.08
**3’,5’-cGMP**	1.78 ± 0.12	0.80 ± 0.31	≥ 0	≤ 0.033 ± 0.006
**3’,5’-cCMP**	2.82 ± 0.28	0.71 ± 0.28	n.d.	n.d.
**3’,5’-cUMP**	2.06 ± 0.32	1.08 ± 0.27	n.d.	n.d.
**3’,5’-cTMP**	n.d.	n.d.	n.d.	n.d.
**3’,5’-cIMP**	n.d.	n.d.	n.d.	n.d.
**3’,5’-cXMP**	n.d.	n.d.	n.d.	n.d.
**2’,3’-cAMP**	151.98 ± 11.22	97.90 ± 17.80	5.02 ± 0.49	3.43 ± 0.94
**2’,3’-cGMP**	105.24 ± 7.70	63.46 ± 13.91	2.66 ± 0.28	2.48 ± 0.71
**2’,3’-cCMP**	82.87 ± 6.53	61.55 ± 9.91	1.19 ± 0.58	1.73 ± 0.48
**2’,3’-cUMP**	2.76 ± 0.62	2.17 ± 0.57	n.d.	≤ 0.13 ± 0.02
**ppGpp**	n.d.	n.d.	n.d.	n.d.
**pppGpp**	n.d.	n.d.	n.d.	n.d.
**3’,5’-c-di-GMP**	n.d.	n.d.	n.d.	n.d.
**3’,5’-c-di-AMP**	20.59 ± 2.03	22.82 ± 1.56	n.d.	n.d.
**5’-pGpG**	n.d.	n.d.	n.d.	n.d.
**5’-pApA**	≤ 1.36 ± 0.19	0.55 ± 0.06	n.d.	≥ 0
**2’,3’-cGAMP**	n.d.	n.d.	n.d.	n.d.
**3’,3’-cGAMP**	n.d.	n.d.	n.d.	n.d.
**3’,2’-cGAMP**	n.d.	n.d.	n.d.	n.d.
**Ap_4A**	3.75 ± 2.25	0.57 ± 0.22	246.90 ± 28.23	16.01 ± 4.51
**c-tetra-AMP**	n.d.	n.d.	n.d.	n.d.

(**±**: gives standard deviation; **n.d.**: not detectable; **≤**: average of all technical replicates ≥ LLOQ; **≥ 0**: nucleotide detected but all technical replicates < LLOQ)

Supplementary References:

1. Allers,T., Ngo,H.-P., Mevarech,M. and Lloyd,R.G. (2004) Development of additional selectable markers for the halophilic archaeon Haloferax volcanii based on the leuB and trpA genes. *Appl. Environ. Microbiol.*, **70**, 943–53.

2. Wagner,M., van Wolferen,M., Wagner,A., Lassak,K., Meyer,B.H., Reimann,J. and Albers,S.-V. (2012) Versatile Genetic Tool Box for the Crenarchaeote Sulfolobus acidocaldarius. *Front. Microbiol.*, **3**, 214.
